# Extensive proteomic and transcriptomic changes quench the TCR/CD3 activation signal of latently HIV-1 infected T cells

**DOI:** 10.1371/journal.ppat.1008748

**Published:** 2021-01-19

**Authors:** Eric Carlin, Braxton Greer, Kelsey Lowman, Alexandra Duverger, Frederic Wagner, David Moylan, Alexander Dalecki, Shekwonya Samuel, Mildred Perez, Steffanie Sabbaj, Olaf Kutsch

**Affiliations:** Department of Medicine, Division of Infectious Disease, University of Alabama at Birmingham, Birmingham, Alabama, United States of America; University of North Carolina at Chapel Hill, UNITED STATES

## Abstract

The biomolecular mechanisms controlling latent HIV-1 infection, despite their importance for the development of a cure for HIV-1 infection, are only partially understood. For example, *ex vivo* studies have recently shown that T cell activation only triggered HIV-1 reactivation in a fraction of the latently infected CD4+ T cell reservoir, but the molecular biology of this phenomenon is unclear. We demonstrate that HIV-1 infection of primary T cells and T cell lines indeed generates a substantial amount of T cell receptor (TCR)/CD3 activation-inert latently infected T cells. RNA-level analysis identified extensive transcriptomic differences between uninfected, TCR/CD3 activation-responsive and -inert T cells, but did not reveal a gene expression signature that could functionally explain TCR/CD3 signaling inertness. Network analysis suggested a largely stochastic nature of these gene expression changes (transcriptomic noise), raising the possibility that widespread gene dysregulation could provide a reactivation threshold by impairing overall signal transduction efficacy. Indeed, compounds that are known to induce genetic noise, such as HDAC inhibitors impeded the ability of TCR/CD3 activation to trigger HIV-1 reactivation. Unlike for transcriptomic data, pathway enrichment analysis based on phospho-proteomic data directly identified an altered TCR signaling motif. Network analysis of this data set identified drug targets that would promote TCR/CD3-mediated HIV-1 reactivation in the fraction of otherwise TCR/CD3-reactivation inert latently HIV-1 infected T cells, regardless of whether the latency models were based on T cell lines or primary T cells. The data emphasize that latent HIV-1 infection is largely the result of extensive, stable biomolecular changes to the signaling network of the host T cells harboring latent HIV-1 infection events. In extension, the data imply that therapeutic restoration of host cell responsiveness prior to the use of any activating stimulus will likely have to be an element of future HIV-1 cure therapies.

## Author summary

A curative therapy for HIV-1 infection will at least require the eradication of a small pool of CD4+ helper T cells in which the virus can persist in an inactive, latent state, even after years of successful antiretroviral therapy. It has been assumed that activation of these viral reservoir T cells will also reactivate the latent virus, which is a prerequisite for the destruction of these cells. Remarkably, this is not always the case and following application of even the most potent stimuli that activate normal T cells through their T cell receptor, a large portion of the latent virus pool remains in a dormant state. Herein we demonstrate that a large part of latent HIV-1 infection events reside in T cells that have been rendered activation inert. We provide a systemwide, biomolecular description of the changes that render latently HIV-1 infected T cells activation inert and using this description, devise pharmacologic interference strategies that render initially activation inert T cells responsive to stimulation. This in turn allows for efficient triggering of HIV-1 reactivation in a large part of the otherwise unresponsive latently HIV-1 infected T cell reservoir.

## Introduction

Antiretroviral therapy (ART) efficiently suppresses HIV-1 replication below detection levels of diagnostic assays, but ART does not eliminate latent viral reservoirs, enabling HIV-1 to persist for the lifetime of a patient and rebound whenever ART is interrupted [1]. The most comprehensive evidence for viral persistence has been presented for latent HIV-1 infection events residing in long-lived, resting CD4 memory T cells [2–4]. Intuitively, this would explain the stability of the latent HIV-1 reservoir, as T cell memory can persist for the life-time of an individual. However, while immunological memory can persist for a life-time, individual memory T cells are relatively short lived. The half-life of individual CD4^+^ central memory T cells (T_CM_ cells) that are thought to serve as host cells of latent HIV-1 infection events ranges between 20 and ~100 days and is generally shorter in HIV patients than in healthy individuals, with most studies suggesting a half-life τ_1/2_ <50 days [5–8]. With an assumed half-life of τ_1/2_ = 50 days and an initial reservoir consisting of 1x10^6^ latently HIV-1 infected CD4^+^ T_CM_ cells, it should take less than three years after the onset of ART for the last latently infected T_CM_ cell to disappear. This is obviously not the case and evidence has been provided that preferential or homeostatic proliferation of latently HIV-1 infected T cells in the absence of reactivation can contribute to the stability of the latent reservoir [9,10]. While homeostatic T cell proliferation must contribute to the stability of the latent HIV-1 reservoir, it is only one contributor to lifelong immunological memory. The second component, repeated exposure to cognate antigen and subsequent re-expansion of the specific memory T cells are also crucial components of lifelong memory maintenance. How the latent HIV-1 infection pool in the memory CD4^+^ T cell population remains stable despite the expected and likely required exposure of latently HIV-1 infected T cells to their cognate antigen, and the resulting potent biological activation of the host T cells, has not been detailed. Over time, encounters with cognate antigen should cause a continuous contraction of the latent HIV-1 reservoir [11]. The absence of a measurable reservoir decay despite the expected continuous encounter of cognate-antigen and subsequent T cell activation could be explained by the idea that memory T cells hosting latent HIV-1 infection events have been altered to exhibit an activation-inert phenotype. TCR/CD3 activation-inertness would explain the inability of early therapeutic interventions (e.g. IL-2 or anti-CD3 mAb OKT3) to trigger a meaningful decrease in the size of the latent HIV-1 reservoir [12–14]. An activation-inert host cell phenotype would also explain findings that a significant part of the latent HIV-1 reservoir is resistant to *ex vivo* T cell activation, mostly based on impaired host cell signaling pathways, without any requirement for a repressive chromatin environment at the viral LTR, which is often not present, as latent HIV-1 is usually integrated into actively expressed genes [15].

A detailed description of the biomolecular biology of latently HIV-1 infected T cells that are TCR/CD3 activation inert is rather complicated. Their presence can only be indirectly detected by activating T cells from the same donor or cell population-based model of HIV-1 latency with anti-CD3 antibody and a subsequent or parallel activation with a more potent stimulus such as PMA/ionomycin, followed by quantification of the level of the resulting reactivated virus production. Should PMA/ionomycin stimulation result in higher levels of measurable HIV-1 production, the differential between these two activation methods would indicate the presence of a TCR/CD3 activation inert latent reservoir. However, per definition, TCR/CD3-activation inert latently HIV-1 infected T cells cannot be directly identified in any bulk T cell population, neither in *ex vivo* cell material from patients, nor in *in vitro* generated populations of primary T cells or T cell lines holding latently HIV-1 infected cells. Thus, direct biomolecular studies describing the detailed biomolecular baseline phenotype of TCR/CD3 activation-inert host cells of latent HIV-1 infection events can only be done in clonal T cell lines in which the inert phenotype has been established prior to analysis.

In this study we formally demonstrate that TCR/CD3-inertness occurs in population-based *in vitro* models of latent HIV-1 infection that utilize primary T cells and is reproduced in populations of HIV-1 infected long-term T cell line cultures. Following the identification of clonal latently infected T cell lines that were TCR/CD3-(re)activation responsive or inert, we were able to describe the molecular basis of this phenomenon in these models using a systems biology approach that described TCR/CD3-inertness at the transcriptome and proteome level. We further demonstrate that network analysis of these data can be used to identify drug targets that can be addressed to restore TCR/CD3 responsiveness. Strikingly, genome-wide RNA-level analysis failed to identify the underlying biomolecular phenotype, which was efficiently detected by proteomic analysis. In either case, the data suggest that stable changes to the biomolecular host cell environment are key to TCR/CD3 reactivation inertness and we discuss the implications of these findings for the development of therapeutic approaches to potentially eradicate the latent HIV-1 reservoir.

## Results

### HIV-1 infection generates a TCR/CD3 activation inert population of latently infected T cells

**Fig 1:**
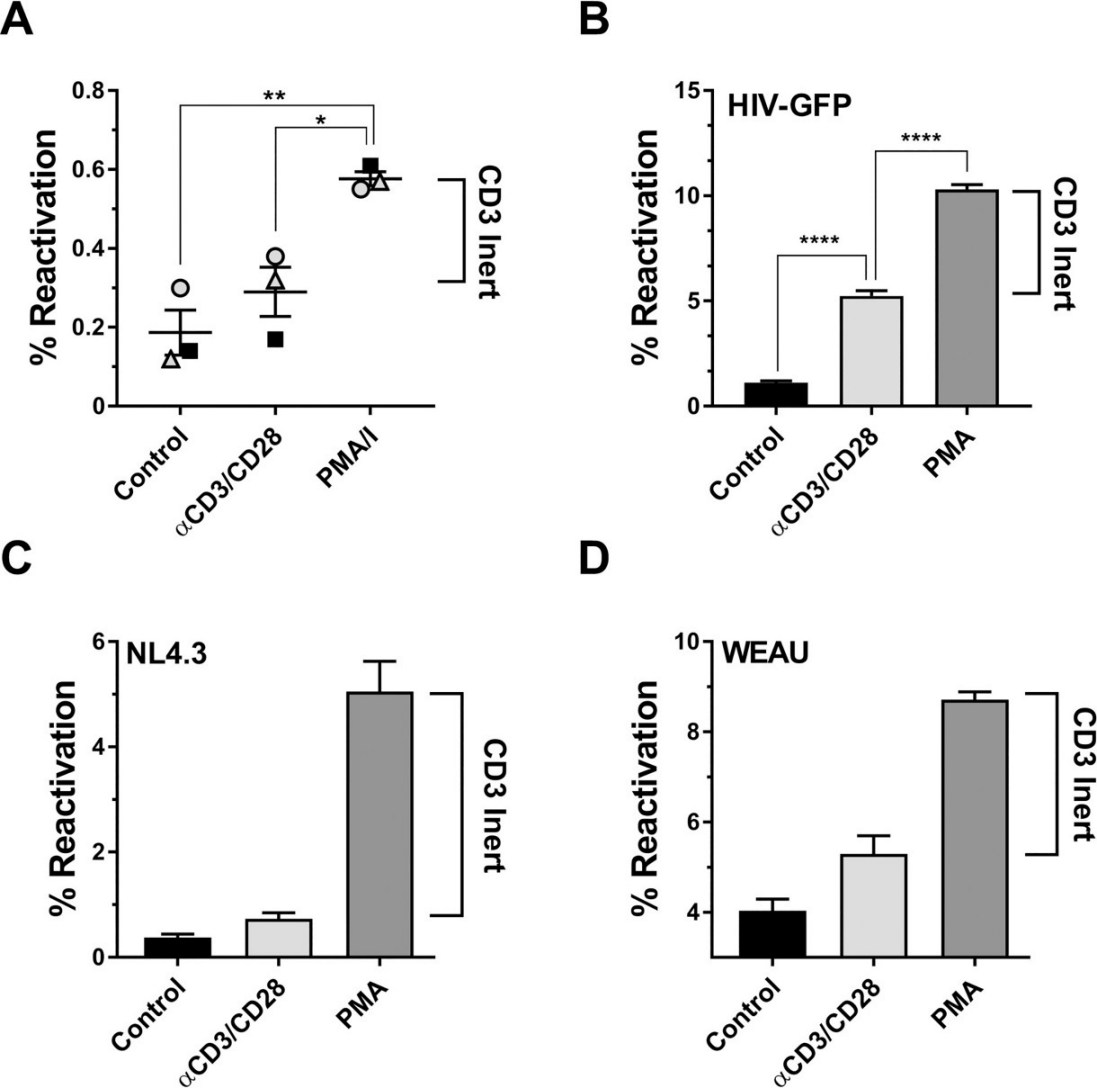
HIV-1 infection induced TCR/CD3 activation-inertness is conserved between primary T cells and T cell lines. (**A**) *In vitro* latently HIV-1 infected primary CD4+ T cells were generated as described under Methods using PBMCs from three different donors infected with a full-length GFP-reporter virus. The T cell infection cultures were then left unstimulated (control), stimulated with anti-CD3/CD28 mAb coated beads or stimulated with PMA/ionomycin. Using GFP expression as a surrogate marker, levels of active/reactivated HIV-1 expression were determined 72 hours post stimulation using flow cytometric analysis for GFP expression. The difference between the GFP+ frequencies following PMA/ionomycin stimulation and CD3/CD28 mAb stimulation for each donor represents the TCR/CD3 activation-inert reservoir. (**B**) Jurkat T cells were infected with a HIV-1 NL4-3 based GFP reporter virus and cultured for a total of 60 days in the presence of RT inhibitors until a stable viral reservoir had been established as previously described [29,30]. At this time, samples of the infection cultures were either left unstimulated, treated with anti-CD3/CD28 mAb coated beads or stimulated with PMA. Baseline HIV-1 expression levels in the untreated cells and HIV-1 reactivation in the stimulated cultures were determined using flow cytometric analysis for GFP expression. The difference between the GFP+ frequencies following PMA stimulation and CD3/CD28 mAb stimulation for each donor represents the TCR/CD3 activation-inert reservoir. Similar experiments were performed using a reporter T cell line that expresses GFP when actively HIV-1 infected (J-R5D4 cells). J-R5D4 T cells were infected with (**C**) HIV-1 NL4-3 or (**D**) HIV-1 WEAU, a primary HIV-1 patient isolate. Following six weeks of culture in the presence of RT inhibitors reactivation experiments were performed as in (B) and reactivation levels were determined by flow cytometric analysis for GFP expression. Data plotted as mean ± standard deviation of three independent experiments.

Previous reports had provided evidence that a large part of the latent HIV-1 reservoir would be recalcitrant to T cell activation, including stimulation of the TCR/CD3 pathway [15,16]. We initially sought to confirm this phenomenon in a well-established primary T cell model of HIV-1 latency [16–22]. Briefly, activated CD4+ T cells from healthy donors were infected with a full length, replication competent HIV-GFP reporter virus and RT inhibitors were added beginning 72hr post infection. At four weeks post infection, the cell cultures were sorted to remove all actively infected and therefore GFP-positive T cells to lower the signal background. The sorted CD4+T cells were then left unstimulated, stimulated with anti-CD3/CD28 mAbs or stimulated with PMA/ionomycin. The latter is widely considered the most potent experimental means to trigger activation and proliferation in primary T cells while completely bypassing the TCR/CD3/CD28 pathway. In line with the idea that a fraction of the host T cells of latent HIV-1 infection events had been rendered inert to TCR/CD3 activation, PMA/ionomycin stimulation of CD4+ T cell HIV-1 infected cultures consistently triggered higher levels of HIV-1 reactivation levels than antibody mediated CD3/CD28 stimulation (Fig 1A).

While these results formally demonstrate that HIV-1 infection establishes both, a TCR/CD3 activation-responsive and a TCR/CD3 activation-inert latent viral reservoir in primary T cells, the model would not lend itself to biomolecular analysis of the observed reactivation inertness phenomenon. To directly describe the biomolecular baseline phenotype of TCR/CD3 activation-inert latently HIV-1 infected T cells it would be necessary to isolate TCR/CD3-responsive and TCR/CD3-inert T cells from these cultures without applying any manipulation to the cells. In the absence of any accepted marker that identifies latently HIV-1 infected primary T cells, or markers for TCR/CD3 reactivation-responsive or TCR/CD3-inert latently HIV-1 infected T cells, direct identification of TCR/CD3 activation-inert T cells within a population is mechanistically impossible. The only possible strategy to accomplish this goal is thus the generation of latently HIV-1 infected T cell clones (based on PMA stimulation), followed by a second round of characterization for TCR/CD3 responsiveness (anti-CD3/CD28 mAb stimulation). This approach requires that HIV-1 infection of T cell lines also generates a separation into TCR/CD3-responsive and TCR/CD3-inert latently HIV-1 infected T cell populations as observed for primary T cells.

Jurkat T cells were early on used to decipher the fundamental molecular biology of TCR/CD3 signaling [23–26] and are now a commonly used CD4+ T cell line to study HIV-1 latency [27–29], making them an ideal system to investigate TCR/CD3 reactivation response in the context of HIV-1 infection. Similar to previous efforts, we infected Jurkat T cells with a replication competent full-length HIV-GFP reporter virus and followed the establishment of latent HIV-1 infection at the population level [18,19,28–31]. After 6 weeks of culture in the presence of RT inhibitors, active background infection was reduced to <2% (control) and addition of PMA triggered reactivation in ~8% of the T cell population over this background (Fig 1B). Antibody mediated stimulation of CD3/CD28 only triggered HIV-1 reactivation in an additional ~3% of the cells over background, suggesting the presence of TCR/CD3-inert latently HIV-1 infected T cells consisting of 5% of the total cell population or ~60% of the overall latently HIV-1 infected subpopulation. This phenomenon could be reproduced in a GFP-reporter T cell population (J-R5D4 cells [30]) that were infected with either the recombinant clone HIV-1 NL43 (Fig 1C) or with HIV-1 WEAU, a primary R5-tropic patient isolate (Fig 1D) [30,32]. In these cells, Tat protein derived following reactivation of the integrated latent full-length virus is required to *in trans* drive the expression of an integrated LTR-gag-pol-GFP-LTR reporter vector. Here, PMA induced 10-fold or 3-fold more reactivation than CD3/CD28 stimulation, indicating that the phenomenon of HIV-1 infection induced TCR/CD3 reactivation that we observed in primary T cells is effectively reproduced in T cell lines. This finding provided us with the ability to generate relevant T cell clones that represent inert and responsive phenotypes.

### Generation of TCR/CD3 activation-responsive and -inert latently HIV-1 infected T cell clones

**Fig 2:**
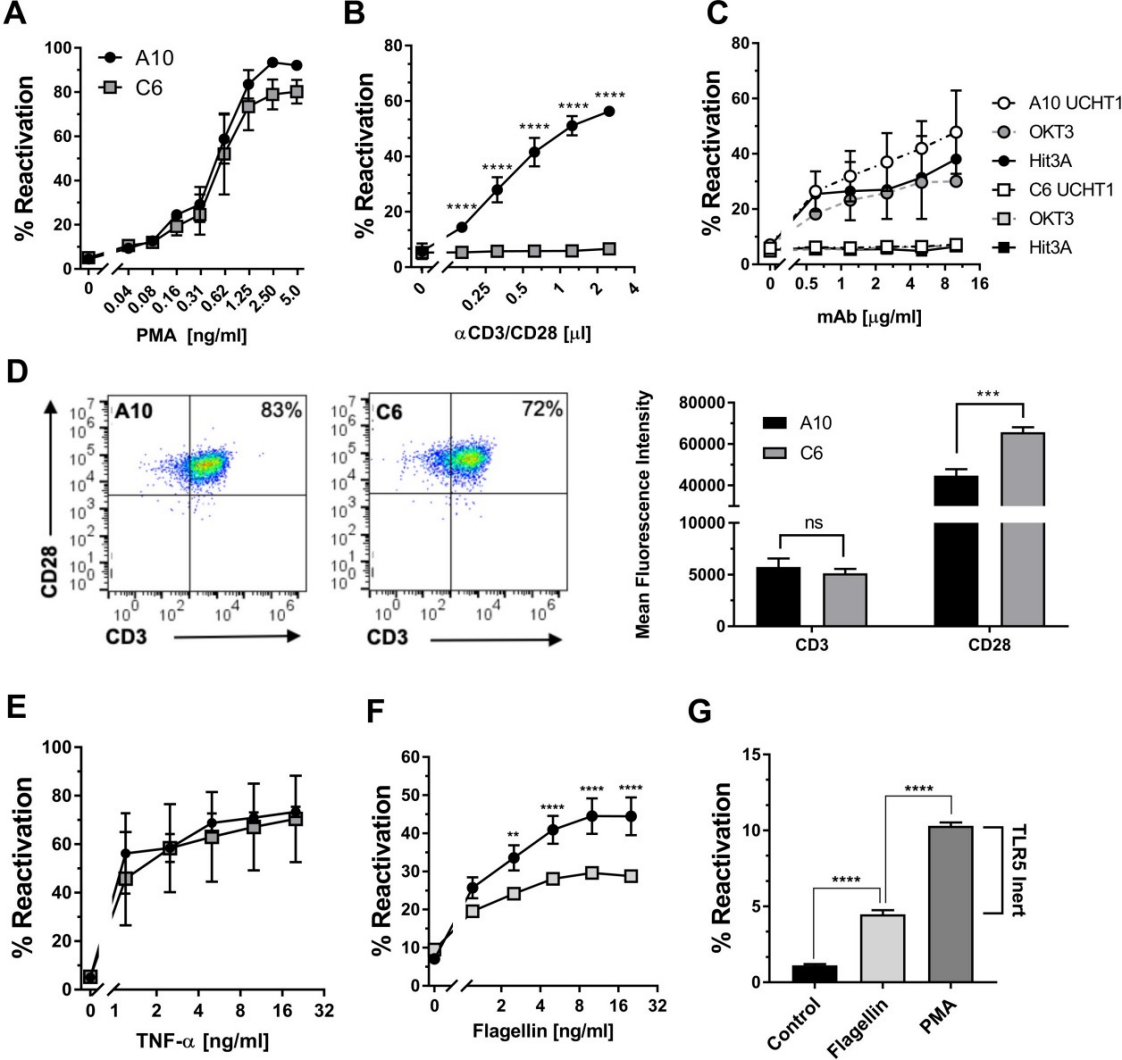
Generation of TCR/CD3-inert and -responsive latently HIV-1 infected T cell clones. (**A**) HIV-1 reactivation levels in JWEAU-A10 and JWEAU-C6 T cells following stimulation with increasing concentrations of the PKC/NF-κB activating phorbolester PMA as determined by flow cytometric analysis for GFP expression. (**B**) HIV-1 reactivation levels in JWEAU-A10 and JWEAU-C6 T cells following stimulation with increasing concentrations of α-CD3/CD28 mAb as determined by flow cytometric analysis for GFP expression. (**C**) HIV-1 reactivation levels in JWEAU-A10 and JWEAU-C6 T cells following stimulation with increasing concentrations of different α-CD3 mAbs (OKT3, UCHT1, HIT3A) as determined by flow cytometric analysis for GFP expression. (**D**) Expression levels of CD3 and CD28 proteins on JWEAU-A10 and JWEAU-C6 T cells as determined by flow cytometric analysis. (**E**) HIV-1 reactivation levels in JWEAU-A10 and JWEAU-C6 T cells following stimulation with increasing concentrations of TNF-α. (**F**) HIV-1 reactivation levels in JWEAU-A10 and JWEAU-C6 T cells following stimulation with increasing concentrations of the TLR5 agonist flagellin. (**G**) HIV-1 reactivation levels in a long-term HIV-1 infected T cell population holding a 10% latently infected subpopulation following stimulation with optimal concentrations of the TLR5 agonist flagellin or PMA. Where indicated, data represent the mean ± standard deviation of at least three independent experiments.

We used the infection culture shown in Fig 1D to generate latently HIV-1 infected T cell clones that would represent TCR/CD3-responsive (JWEAU-A10) and TCR/CD3-inert (JWEAU-C6) T cells. In these T cell clones, PMA triggered an efficient HIV-1 reactivation response (Fig 2A), but even saturating amounts of CD3/CD28 mAbs that triggered reactivation in ~60% of JWEAU-A10 T cells, would not trigger HIV-1 reactivation in JWEAU-C6 cells. (Figs 2B and S1). TCR/CD3-reactivation inertness in JWEAU-C6 T cells was not a function of the utilized antibody clone (OKT3, UCHT1, HIT3A) (Fig 2C) and flow cytometric analysis demonstrated that TCR/CD3 inertness was also not the result of reduced levels of CD3 or CD28 expression on JWEAU-C6 (Fig 2D).

TNF-α, which also triggered NF-κB activation, albeit through a different upstream pathway, would induce similar levels of HIV-1 reactivation in JWEAU-A10 and JWEAU-C6 T cells (~65%; Fig 2E). Bacterial flagellin, another NF-κB agonist that signals through toll-like receptor 5 (TLR5), triggered reduced levels of HIV-1 reactivation in JWEWAU-A10 T cells (~45%) and was further compromised in JWEAU-C6 T cells (<30%) (Fig 2F). A diminished flagellin-induced reactivation response was also observed at the population level, where two-thirds of the latent population remained unresponsive to TLR5 activation (Fig 2G). Differences in the host cell signaling networks that promote latent HIV-1 infection and restrict reactivation are thus not limited to the TCR/CD3 signaling pathway and extend to other activating receptors/pathways that are currently investigated as targets of latency reversing agents (LRA) [33–37].

**Fig 3:**
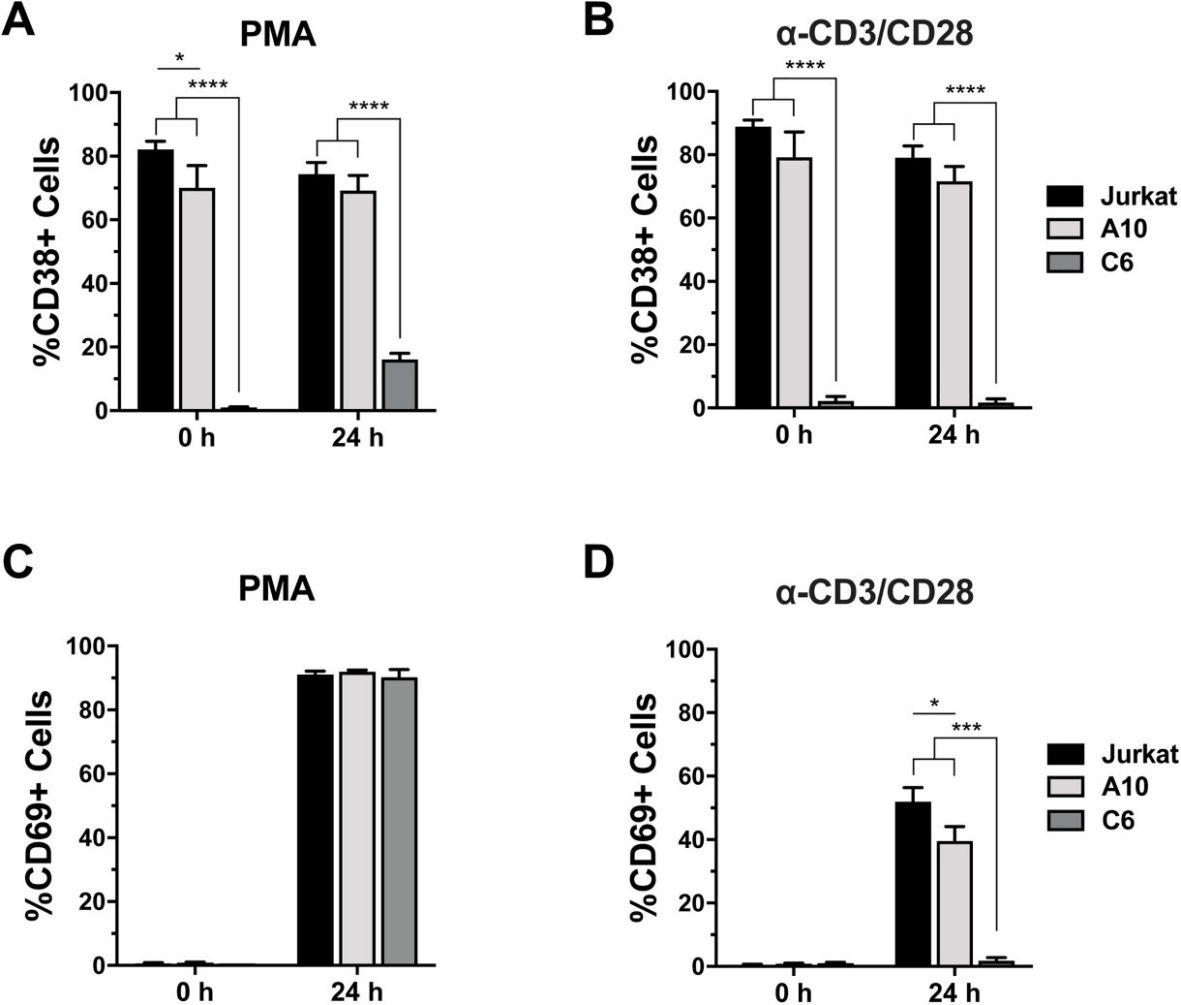
Regulation of T cell activation markers on TCR/CD3-inert and -responsive latently HIV-1 infected T cell clones. (**A**) CD38 expression on uninfected Jurkat, JWEAU-A10 and JWEAU-C6 T cells was determined at baseline and 24 hours post PMA stimulation using flow cytometric analysis. (**B**) CD38 expression on uninfected Jurkat, JWEAU-A10 and JWEAU-C6 T cells was determined at baseline and 24 hours post α-CD3/CD28 mAb stimulation using flow cytometric analysis. (**C**) CD69 expression on uninfected Jurkat, JWEAU-A10 and JWEAU-C6 T cells was determined at baseline and 24 hours post PMA stimulation using flow cytometric analysis. (**D**) CD69 expression on uninfected Jurkat, JWEAU-A10 and JWEAU-C6 T cells was determined at baseline and 24 hours post α-CD3/CD28 mAb stimulation using flow cytometric analysis. Data represent the mean ± standard deviation of at three independent experiments.

Analysis of the T cell activation markers CD38 and CD69 at baseline and following stimulation revealed additional insights into the fundamental differences between the responsive JWEAU-A10 and the inert JWEAU-C6. CD38, a type II glycoprotein that marks T cells with an activated phenotype [38] was highly expressed already at baseline on Jurkat T cells and the responsive JWEAU-A10 T cells, but not on the activation-inert JWEAU-C6 T cells (Fig 3A). Stimulation with PMA (Fig 3A) or anti-CD3/CD28 mAbs (Fig 3B) only triggered minimal or no induction of CD38 expression on JWEAU-C6 T cells. CD69, an immediate early T cell activation marker at baseline was absent on all three cell types, but upon PMA activation was efficiently upregulated and expressed on >80% of all cells, with no discernable difference between the three cell lines (Fig 3C) [39,40]. CD3/CD28 mAb stimulation triggered CD69 upregulation in ~50% of Jurkat and ~40% of JWEAU-A10 T cells, but completely failed to trigger CD69 expression in JWEAU-C6 T cells (Fig 3D). As CD69 expression, similar to HIV-1 or CD38 expression, is driven by NF-κB family transcription factors [41], these data show that CD3/CD28 reactivation inertness is not specific to viral transcriptional control, but reflective of a general cellular CD3/CD28 activation inertness in JWEAU-C6 T cells. The data further indicate that the activation inert JWEAU-C6 are characterized by an already lower baseline activation state compared to their activation responsive counterparts JWEAU-A10.

### CD3 stimulation fails to generate a NF-κB signal in inert latently HIV-1 infected T cells

**Fig 4:**
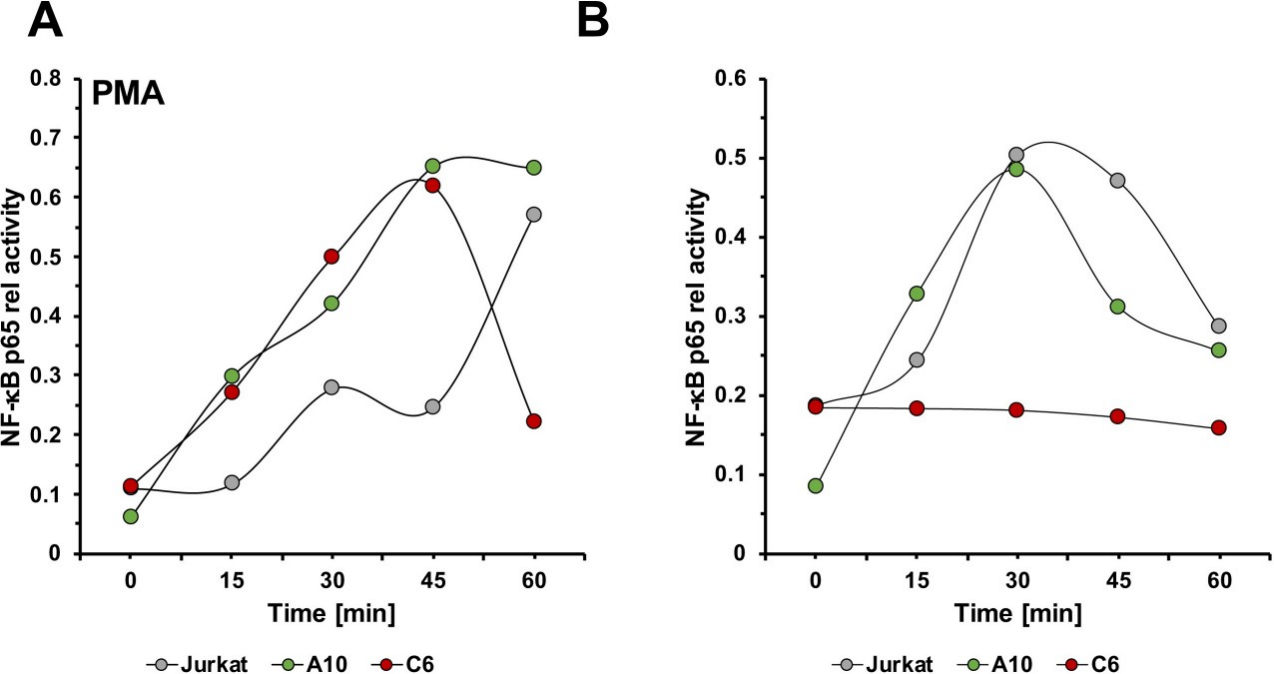
NF-κB activation kinetics in TCR/CD3-inert and -responsive latently HIV-1 infected T cell clones. Uninfected Jurkat, JWEAU-A10 and JWEAU-C6 T cells were stimulated with either (**A**) PMA or (**B**) α-CD3/CD28 mAbs and sampled at the indicated timepoints. Phospho-p65 NF-kB signals were quantified with a TransAM ELISA and plotted over a time axis.

Given that different NF-κB agonists, such as PMA, TNF-α or flagellin efficiently or at least partially triggered HIV-1 reactivation in JWEAU-C6 T cells, it stands to reason that TCR/CD3 reactivation inertness in these cells is not the result of a generally dysfunctional canonical NF-κB pathway, but a phenomenon that is specific for the TCR/CD3 signaling pathway. To detail the kinetic NF-κB response in the two latently HIV-1 infected T cell lines relative to uninfected Jurkat T cells, we performed TransAM assays measuring NF-κB p65 phosphorylation 15, 30, 45, and 60 minutes post PMA or anti-CD3/CD28 mAb stimulation (Fig 4). Interestingly, despite the ability to induce efficient HIV-1 reactivation, the kinetic NF-κB signal induced by PMA in either latently HIV-1 infected T cell line already differed from the signal in uninfected T cells. Whereas PMA stimulation produced the expected sinus-wave shaped signal with increasing amplitude in the uninfected control cells over the monitored 60 minutes time period [42], PMA stimulation resulted in a single high-amplitude peak in both latently HIV-1 infected T cells, that within the 60 min observation period returned to baseline in JWEAU-C6 T cells, but not in JWEAU-A10 T cells (Fig 4A). The CD69 expression data shown in Fig 3C are derived from this experiment, demonstrating that stimulation was equally successful for al cell types. CD3/CD28 stimulation triggered identically shaped kinetic NF-κB activation response curves in Jurkat and JWEAU-A10 T cells, which for Jurkat T cells differed from the PMA-induced activation pattern (Fig 4B). Both cell types produced a single peak signal in the 60 min observation period, with Jurkat T cells possibly showing a slightly more extended signal response. In the inert JWEAU-C6 T cells the ability of CD3/CD28 stimulation to induce a NF-κB signal was completely abrogated, conclusively explaining the inability of TCR/CD3 activation to trigger HIV-1 reactivation.

### Gene expression patterns associated with TCR/CD3 activation-inert latently HIV-1 infected T cells

To identify the biomolecular basis of TCR/CD3 activation inertness, we first generated RNA-seq data describing the gene expression signatures of uninfected T cells, the TCR/CD3-inert JWEAU-C6 T cells and the TCR/CD3-responsive JWEAU-A10 T cells. A total of 7,151 genes (of 20,830 genes with any reads) were differentially expressed (likelihood ratio test, adjusted *P*-value < 0.01) across the three cell types. Pathway analysis of the complete mRNA-level data set did not produce any information that would suggest impairment of the TCR signaling or any related pathway, which was surprising given that this was the predetermined phenotype. In fact, pathway enrichment analysis produced a series of completely unrelated motifs the highest ranked being cardiac development (3.74E-03) and leukocyte chemotaxis (3.77E-03). Also, despite the high number of input genes, all motifs were suggested with very low confidence (S1 Table).

**Fig 5:**
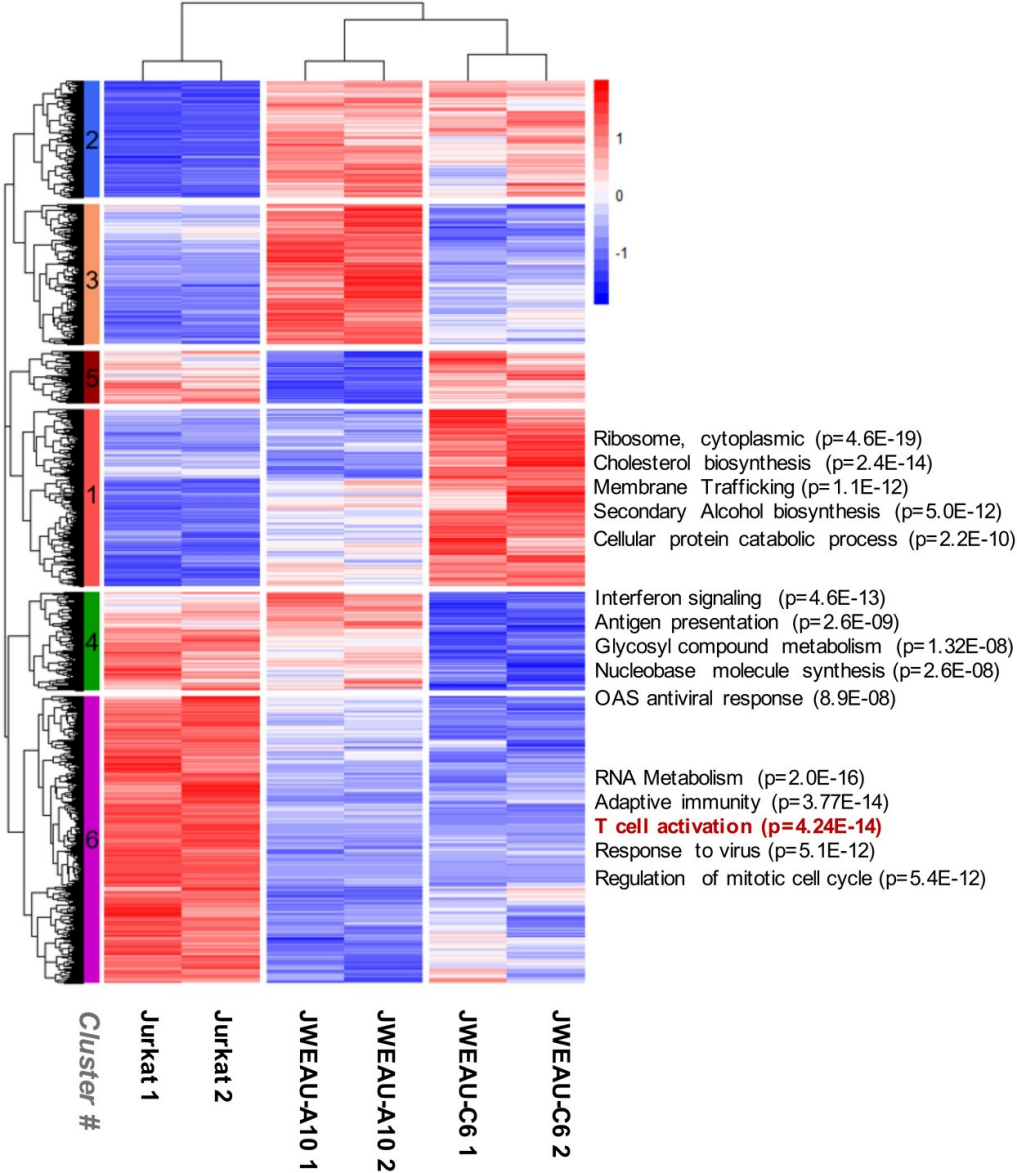
Transcriptomic analysis reveals extensive differences between TCR/CD3-inert and -responsive latently HIV-1 infected T cell clones. RNA-seq analysis was used to describe the genome-wide RNA expression signature of uninfected Jurkat T cells, the TCR/CD3 responsive JWEAU-A10 and the TCR/CD3-inert JWEAU-C6 T cells. For each T cell line, RNA-seq data were obtained for low and high cell density conditions. 7,151 genes with differential expression (likelihood ratio test, adjusted *P*-value < 0.01) across the three cell types were subjected to hierarchical clustering. Subtrees representing genes that were lowly or highly expressed throughout all three cells were automatically omitted through the selection criteria. Results for motif enrichment analysis for clusters #1, #4 and #6 are presented.

We reasoned that hierarchical clustering of the gene expression data followed by cluster-based pathway enrichment analysis could improve the likelihood of discovering motifs that control latency and TCR/CD3 reactivation inertness (Fig 5). For two of the six clusters, regulation of genes was largely shared between the two latently HIV-1 infected T cell lines, but different from the parental T cells (cluster #2 (960 genes) and cluster #6 (2353 genes)). We would expect these clusters to contain motifs that are associated with a general latency phenotype. Gene Ontology (GO) enrichment analysis of cluster #2 genes (upregulated in latently HIV-1 infected T cells) suggested changes to lipid metabolism, regulation of cellular component size, and actin filament-based processes [43]. Cluster #6 genes were reported as critical to RNA metabolism, adaptive immune system function, the antiviral response, regulation of the mitotic cell cycle, and, interestingly, T cell activation (Fig 5) (for more details see S2 Fig). Many of these motifs have been associated with HIV-1 latency, but the data would suggest that TCR signaling pathway impairment is a general feature of latently HIV-1 infected T cells and that the magnitude of changes to this pathway is variable. TCR/CD3 reactivation inertness would then only be the most extreme phenotype on a spectrum of possible inertness phenotypes, which may be supported by the data that even optimal anti-CD3/CD28 mAb stimulation remains inferior to PMA activation and only triggers up to 70% reactivation in JWEAU-A10 T cells.

In the remaining four clusters, gene regulation differed between the two latently HIV-1 infected T cells, but one or the other had gene regulation patterns similar to the parental T cells (cluster #1 (1449 genes), #3 (1155 genes), #4 (805 genes), #5 (429 genes). We had expected to extract information content regarding the predetermined TCR/CD3-activation inertness from these clusters, but surprisingly, none of the pathway analysis for these clusters suggested critical relevance to CD3-responsiveness (S2 Fig). A focused analysis of the genes involved T cell activation (GO term 0042110) confirmed that there was no significant difference between the two latently HIV-1 infected T cell clones, but both latently infected T cell lines differed in their gene expression signature from uninfected T cells (S3 Fig). Somewhat surprisingly, the RNA-level data only supported the conclusion that TCR/CD3 signaling impairment is a common feature of latently HIV-1 infected T cells, but could not explain the observed TCR/CD3 stimulation response differences.

### Transcriptomic heterogeneity as the result of extensive stochastic changes to gene expression patterns

The extent of the observed transcriptomic shift between the latently HV-1 infected cells was certainly surprising. Of the total observed changes, 3,313 (46%) were shared between both cell lines, but 3,838 (54%) occurred only in one of the latently infected T cell clones. These findings are consistent with recent reports suggesting vast heterogeneity between individual latently HIV-1 infected T cells and emphasize that HIV-1 latency can be maintained in extensively different cellular environments [44,45]. Mechanistically the data raised the possibility that the extensive amounts of gene expression changes that are not shared between latently HIV-1 infected T cells. Instead, these changes may be stochastic in nature and create an unspecific reactivation threshold by broadly affecting the overall signal transduction ability of host cells of latent HIV-1 infection events.

To explore the idea that the changes to the transcriptome were mostly stochastic in nature, we first tested whether network analysis software would be able to efficiently link genes with altered expression into a functional network. Specifically, MetaCore network analysis software can build protein interaction networks based on reported molecular interactions collected in a manually curated database consisting of information derived from ~1 million peer reviewed publications. Based on the vast extent of curated interactions spanning from transcriptional regulation to post translational modifications, MetaCore software should be expected to efficiently recognize possible links between altered signals from our data set. A high degree of linkage in the network would indicate a functionally interlinked nature of the genes with altered expression, whereas low linkage would suggest stochastic gene regulation effects.

To avoid overloading the network software, we condensed the data set by performing more stringent pairwise comparison between the parental Jurkat T cells and each of the latently HIV-1 infected T cell clones. Significant signals were defined to have a padj < 0.01, a fold-change ≥2; and the read count for at least one the cell types had to be >250. As expected, this process did not alter the proportion of shared (45%, 626 genes) and non-shared genes (55%) between the two latently infected T cell lines, relative to the initial analysis (Fig 5).

A direct interaction algorithm, which builds a protein-protein interaction network exclusively used the genes that were shared between the two latently HIV-1 infected T cell lines, but differed from the uninfected Jurkat T cells as network nodes, only integrated 53% of the seed nodes. Especially, as these were the altered genes that were shared between the two latently infected T cell lines and that would have to be responsible for a common latency control motif (as opposed to non-shared genes that would immediately seem more randomly expressed) this low linkage rate would suggest a very low degree of controlled gene regulation events in the data set (S4 Fig). 50% of the linked genes (293 genes) in the network were connected with only a single link meaning that their regulation were endpoint events and did not contribute to any network functionality (S4 Fig). Only 8 genes had more 20 or more links with other genes. On average each node of the network had only three interactions. Both the failure to efficiently link all altered signals into the network and the low level of interactions per node suggest that a large portion of the observed gene expression changes are not functionally related and represent stochastic gene regulation events, possibly triggered by cellular mechanisms/pathways induced by the actual infection event. We will henceforth refer to this extensive group of functionally unconnected, stochastic gene regulation events as transcriptomic noise. Largely stochastic gene regulation effects also would conclusively explain why motif enrichment analysis of the transcriptomic data set produced only T cell unrelated motifs with very low statistical confidence (S1 Table).

### Validating a role of transcriptomic noise as reactivation threshold for TCR/CD3 signaling

**Fig 6:**
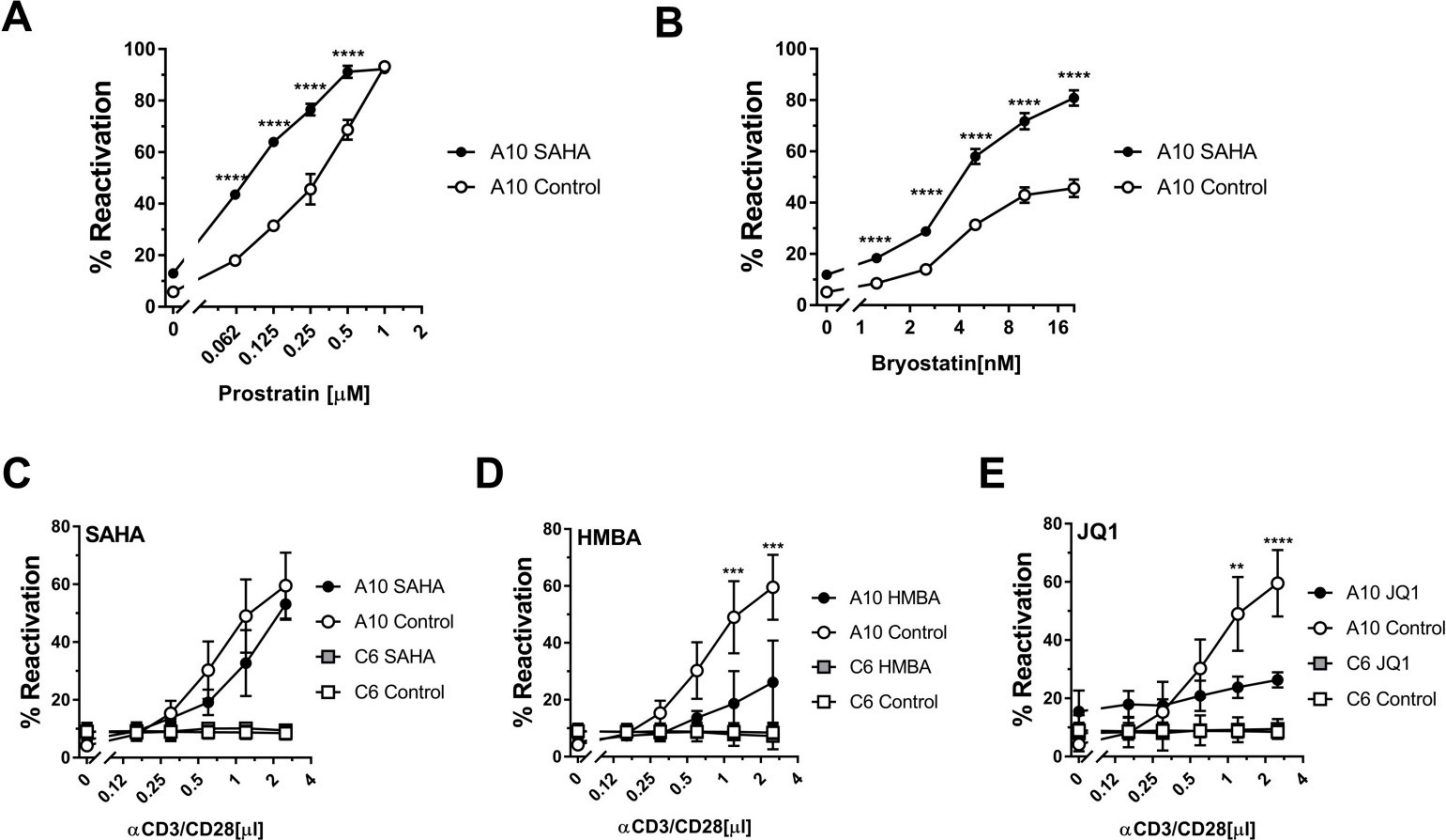
Induction of genetic noise suppresses TCR/CD3 induced HIV-1 reactivation. To determine if transcriptomic noise contributes to the stability of latent HIV-1 infection we pretreated the TCR/CD3 responsive JWEAU-A10 and the TCR/CD3 inert JWEAU-C6 T cells with compounds known to induce transcriptomic noise by promoting unspecific transcriptional elongation and then stimulated the cells with either PMA or α-CD3/CD28 mAbs. To confirm that JWEAU-A10 T cells reproduce previous findings by Dah et al. the cells were treated for overnight with SAHA and then stimulated with (**A**) increasing concentrations of prostratin or (**B**) bryostatin. Cells were treated overnight with (**C**) the HDAC inhibitor SAHA (300 nM), (**D**) the cell differentiating agent HMBA (2mM), or (**E**) the BET inhibitor JQ1 (10μM) and then treated with increasing concentrations of α-CD3/CD28 mAbs. HIV-1 reactivation levels were determined by flow cytometry for GFP expression 24 hours post CD3/CD28 activation. Data represent the mean ± standard deviation of three independent experiments.

Outside the field of HIV-1 research, histone deacetylase (HDAC) inhibitors, BET protein inhibitors or cell differentiating agents are well known to trigger genome-wide changes to gene expression patterns, through a variety of specific and off-target effects, and as such create transcriptomic noise [46–56]. In the context of HIV-1 latency research Dah *et al*. have demonstrated that HDAC inhibitors would trigger or facilitate HIV-1 reactivation by inducing an increase in genetic noise [57] and the varying potential of different histone deacetylase inhibitors to trigger HIV-1 reactivation has been linked to the induction of differential host cell responses [58]. To confirm that our experimental system would reproduce the findings by Dah et al., we initially tested the effect of the histone deacetylase inhibitor suberanilohydroxamic acid (SAHA; vorinostat) on PKC agonist mediated HIV-1 reactivation. As described by Dah et al. pretreatment with SAHA increased the ability of specifically lower concentrations of prostratin and bryostatin to trigger HIV-1 infection in JWEAU-A10 T cells (Figs 6A and 3B). Having confirmed the experimental findings by Dah *et al*., we reasoned that if transcriptomic noise contributes to a TCR/CD3 activation threshold in latently HIV-1 infected T cells, then treatment of the TCR/CD3-responsive JWEAU-A10 T cells with HDAC inhibitors, BET inhibitors or cell differentiating agents, would increase the level of transcriptomic noise, but in contrast to what was observed for PKC agonists, would reduce the ability of TCR/CD3 activation to trigger HIV-1 reactivation.

Following a 24 hours pretreatment period to allow each inhibitor to exert its full effect, non-toxic concentrations of the histone deacetylase inhibitor SAHA had no direct effect on HIV-1 reactivation in either, JWEAU-A10 and JWEAU-C6 T cells (Fig 6C). As predicted, SAHA decreased the TCR/CD3 stimulation-induced HIV-1 reactivation response of JWEAU-A10 T cells, while not restoring a TCR/CD3 response in JWEAU-C6 T cells. The cell differentiating compound hexamethylene bisacetamide (HMBA), which has been reported to trigger HIV-1 reactivation in some experimental systems [59,60], did not directly trigger HIV-1 reactivation (Fig 6D). However, in line with its ability to induce transcriptomic noise HMBA suppressed the TCR/CD3 stimulation-induced HIV-1 reactivation response of JWEAU-A10 T cells. HMBA had no effect on latent HIV-1 infection in JWEAU-C6 T cells, neither by itself nor following TCR/CD3 stimulation. Lastly, we tested the effect of JQ1, a Brd4 inhibitor that has been reported to not only affect global cellular gene expression, but to trigger low level HIV-1 reactivation [61–63]. JQ1 triggered modest levels of HIV-1 reactivation by itself in JWEAU-A10 T cells, but completely suppressed the ability of TCR/CD3 stimulation to trigger any additional HIV-1 reactivation (Fig 6E). As each of these compounds is a documented LRA, their inhibitory effect on TCR/CD3 mediated HIV-1 reactivation of necessity cannot come from interactions of the compounds with functionalities on the HIV-1 LTR, but must be caused by the reported ability of these compounds to trigger transcriptomic noise that affects the CD3 signaling efficacy. As such, the findings indirectly validate a role for transcriptomic noise as a factor affecting TCR/CD3 activation mediated HIV-1 reactivation.

### Kinomic description of CD3-activation inert latently HIV-1 infected T cells

**Fig 7:**
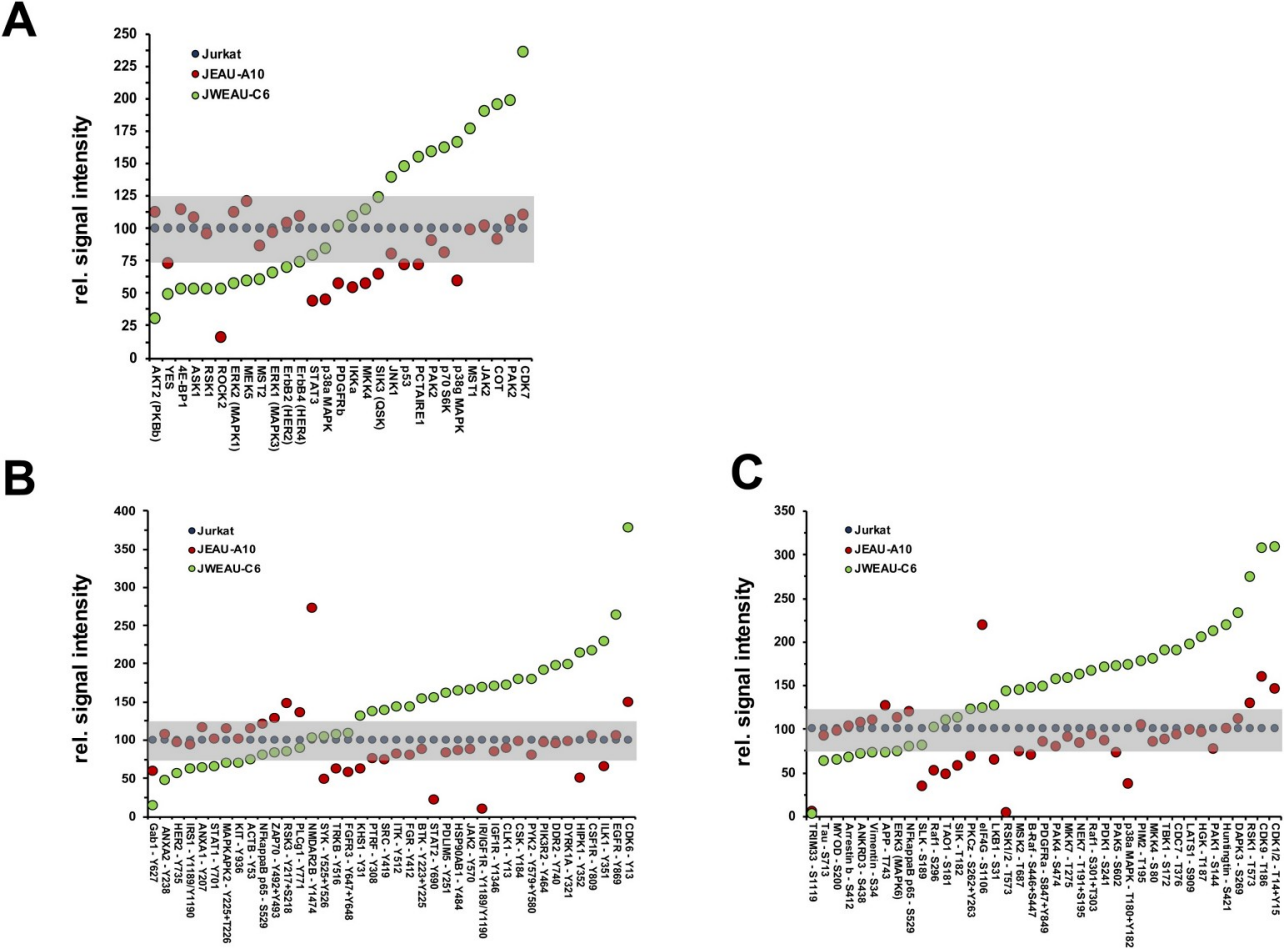
Kinomic analysis of TCR/CD3-inert and -responsive latently HIV-1 infected T cell clones. Cell lysates from JWEAU-A10 (responsive) and JWEAU-C6 T cells (inert) were loaded on antibody array chips (Kinexus KAM-900) to determine differences in protein expression and protein phosphorylation. Each of the two identical arrays on the chip is spotted with 265 pan-specific antibodies and 613 phosphosite specific antibodies. (**A**) Protein expression signals that differed in JWEAU-A10 or JWEAU-C6 T cells from Jurkat T cells were plotted as relative signal percentage normalized to expression in uninfected Jurkat T cells. Signals were ordered based on their relative expression in the activation-inert JWEAU-C6 T cells. Signals in the gray zone (max. 25% deviation from controls) would be considered not altered. (**B**) Tyrosine phosphorylation signals that differed in JWEAU-A10 or JWEAU-C6 T cells from Jurkat T cells were plotted as relative signal percentage normalized to expression in uninfected Jurkat T cells. Signals were ordered based on their relative signal intensity in the activation-inert JWEAU-C6 T cells. (**B**) Serine-threonine phosphorylation signals that differed in JWEAU-A10 or JWEAU-C6 T cells from Jurkat T cells were plotted as relative signal percentage normalized to expression in uninfected Jurkat T cells. Signals were ordered based on their relative signal intensity in the activation-inert JWEAU-C6 T cells.

A possible explanation for the inability of the RNA-level analysis to directly identify the underlying functional impairment (TCR/CD3-inertness) that stabilizes latent HIV-1 infection is that major cellular regulation effects often occur at the level of post-transcriptional or even post-translational modifications (e.g. protein phosphorylation). Changes to the proteome at the level of protein expression and phosphorylation would be the most direct indicators of functional modifications of the host cell signaling network that cause TCR/CD3-inertness and contribute to the stability of latent HIV-1 infection. To identify protein regulation effects that differ between TCR/CD3-responsive and TCR/CD3-inert T cells we used antibody arrays (Kinexus KAM-900). Each antibody array was spotted with 265 pan-specific antibodies and 613 phosphosite specific antibodies. Between JWEAU-A10 and JWEAU-C6 T cells the array identified a total of 123 significant differences. 74% of the signals were phospho-site specific and 26% were pan-specific, which is proportional to the bias of the antibody array for phospho-specific antibodies. Relative to the parental Jurkat T cells the majority of these differences were found in the TCR/CD3-inert JWEWAU-C6 T cells, where a total of 58 signals were found upregulated and 29 downregulated relative to Jurkat T cells, as opposed to 10 upregulated and 33 downregulated signals in JWEAU-A10 T cells. A detailed breakdown regarding regulation of protein expression and tyrosine- versus serine/threonine phosphorylation signals relative to uninfected control cells displays the obvious dysregulation of the phospho-kinome, particularly in the inert JWEAU-C6 cells (Fig 7).

**Fig 8:**
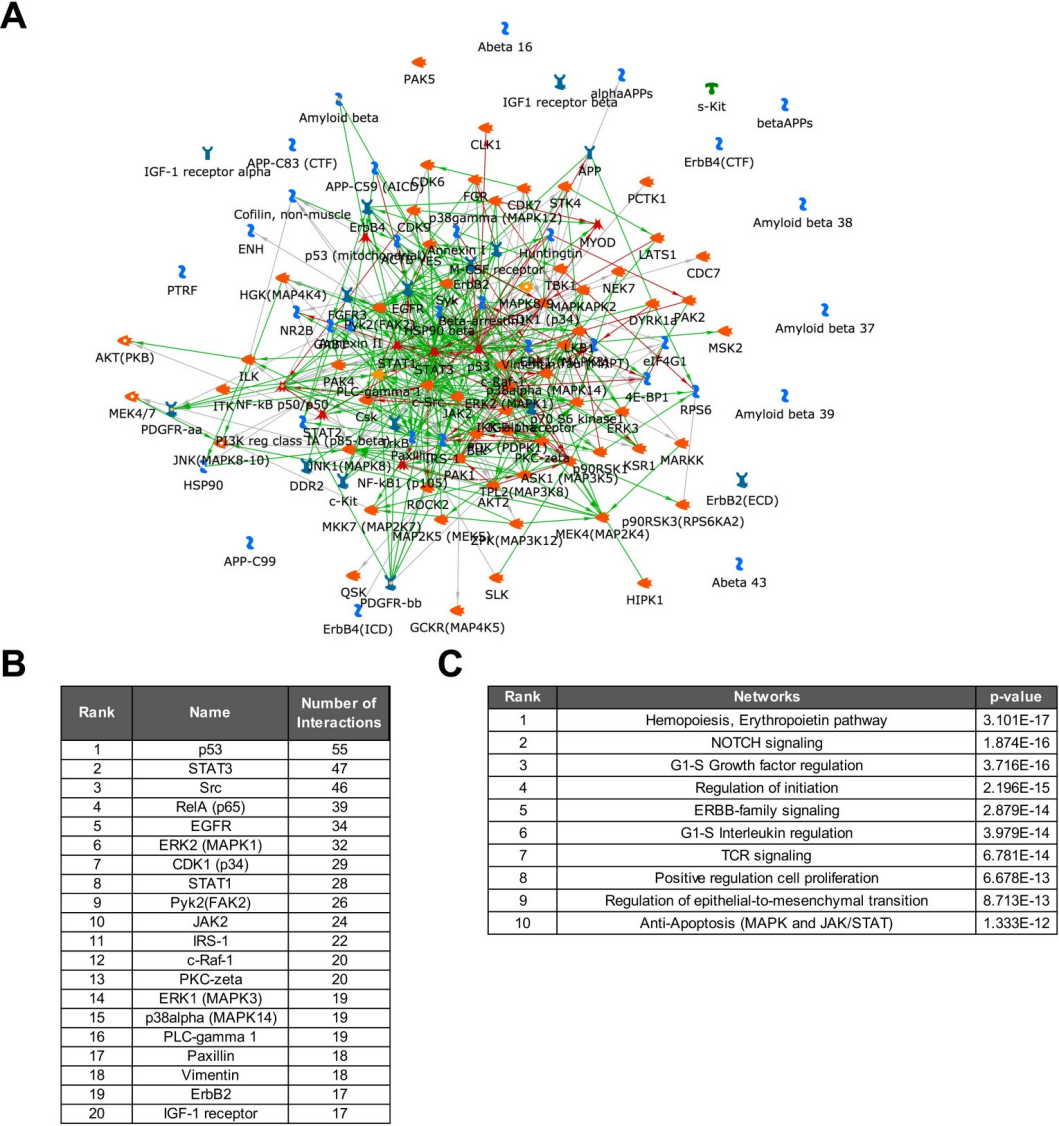
Network analysis of kinomic data describing the biomolecular basis of reactivation inertness in T cells. (**A**) The altered kinomic signals identified between activation responsive JWEAU-A10 and activation-inert JWEAU-C6 T cells were used to generate a protein-protein interaction network using the direct interaction algorithm of MetaCore software. The network visualizes the high level of interactions with few seed nodes (proteins with altered signals) not being connected. (**B**) List of the 20 highest connected altered proteins and their numbers of interactions with other proteins in the network. (**C**) List of the highest ranked functional motifs associated with the network.

Other than for the transcriptomic data, the MetaCore direct interaction algorithm generated an efficient network from these differentially regulated signals. 86% of the signals were linked into a direct protein-protein interaction network, which had an average degree of 14 interactions per node, a sign that likely more than one pathway within the overall network was affected, and no single master switch controlled the recorded changes (Fig 8A). The three highest linked nodes in the network were p53 (55 interactions), STAT3 (47 interactions), and c-Src (46 interactions) (Fig 8B). Also, consistent with the functional phenotype, pathway enrichment analysis identified *Immune Response/TCR signaling* (p = 6.78E-14) as a highly ranked altered motif in addition to other motifs that could be reasonably relevant for HIV-1 latency control (G1-S growth factor regulation, 3.716E-16; regulation of initiation, 2.196E-15; NOTCH signaling, 1.874E-16) (Fig 8C).

**Fig 9:**
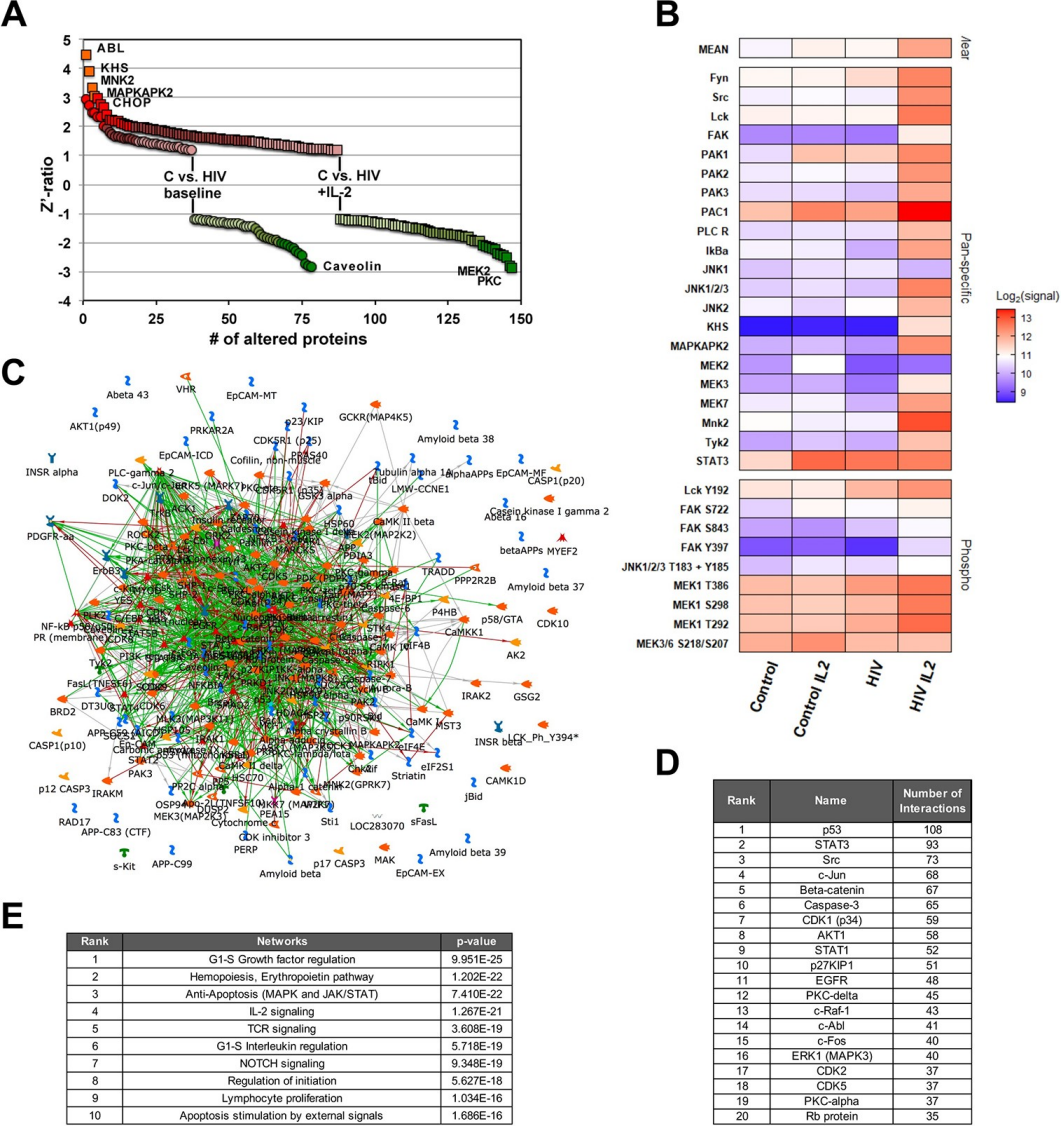
T cells from HIV-1 patients on ART exhibit an altered kinomic phenotype. (**A**) Kinome array analysis was performed using T cells from 9 healthy individuals and 8 HIV/ART patients. Samples were produced either at baseline or following 24h of IL-2 incubation. The samples for each experimental conditions were pooled to minimize individual variations and loaded onto Kinexus antibody arrays. Signals that were altered in samples from HIV/ART patients relative to healthy controls at baseline or following IL-2 stimulation were plotted. (**B**) Focused analysis of kinase and transcription factor signals from the kinome array involved in the TCR/CD3 signaling pathway represented as a heat map (expression or phosphorylation) for each of the four experimental conditions. (**C**) The altered kinomic signals identified between T cells from healthy individuals and T cells from HIV/ART patients were used to generate a protein-protein interaction network using the direct interaction algorithm of MetaCore software. The network visualizes the high level of interactions with few proteins with altered signals not being connected. (**D**) List of the 20 highest connected altered proteins and their numbers of interactions with other proteins in the network. (**E**) List of the 10 highest ranked functional motifs associated with the network.

Being cognizant of the fact that these data were generated in T cell lines, we chose to generate a second phospho-kinome data set for a primary T cell system that could ultimately guide drug target prioritization among the identified central nodes of the protein-protein interaction network. Obviously, as a result of the scarcity of latently HIV-1 infected T cells *in vivo*, the inability to identify these rare cells in the absence of previous activation, and the complete impossibility to identify TCR/CD3-inert primary T cells, we needed to generate phospho-kinome data using a relevant surrogate T cell population that also provides the required amount of cells (~5x10e6 cells per array). Given the substantial body of literature describing a broadly impaired ability of T cells from HIV/ART patients to respond to stimulation, also after the initiation of ART [64–72], we isolated CD4+ T cells from HIV/ART patients (n = 8) that were on ART for less than 2 years and from healthy controls (n = 11). We chose two experimental conditions, (i) unstimulated and (ii) overnight exposure to IL-2, a simple experimental proxy condition for T cells that would reside in an immunologically more active environment such as lymph nodes. Consistent with the idea that T cells from HIV/ART patients have an altered biomolecular phenotype, under unstimulated conditions, kinome array experiments identified a total of 78 differences (37 up-/41 down-regulated) between T cells from healthy controls and HIV/ART patients, and 147 differences (87 up/60 down) following overnight exposure to IL-2 (Fig 9A). A focused analysis of the data for significantly altered proteins reported to be involved in TCR/CD3 signaling further highlighted the impairment of this pathway in T cells from HIV/ART patients, in particular following IL-2 exposure (Fig 9B). MetaCore driven network analysis integrated 83% of the altered signals into a direct interaction network (Fig 9C). 54 nodes had more than 20 interactions in the network, suggesting an overall high degree of linkage between the nodes and a relatively flat signaling hierarchy. Within this network the central proteins (hubs) were p53 (108 interactions), STAT3 (93 interactions), Src (73 interactions), c-Jun (68 interactions), and ß-catenin (67 interactions), all factors that play key roles in TCR/CD3 signaling (Fig 9D) [73–80]. Pathway enrichment analysis ranked G1-S growth factor regulation (p = 9.91E-25), IL-2 signaling (p = 1.26E-21) and TCR signaling (p = 3.60E-19) in the top five altered network motifs (Fig 9E), showing clear similarities to the changes we observed in latently HIV-1 infected T cells.

These data again validate the ability of kinome analysis in conjunction with MetaCore network analysis software to accurately detect and describe experimental conditions (IL-2 signaling) and phenotypes (TCR signaling) and should provide an efficient tool to guide target identification by providing the ability to distinguish between HIV-1 infection induced changes that are shared by primary and immortalized T cells, and changes that are found in latently HIV-1 infected T cell lines, but tat are not present in primary T cells.

### Probing the role of individual network nodes in CD3 inertness and HIV-1 latency control

Under the assumption that individual, highly linked network nodes act as molecular switches that control latent HIV-1 infection, or are key to TCR/CD3-inertness, we chose three targets that were identified through network analysis (Figs 8 and 9). Targets needed to be (i) central nodes in the network describing the kinomic differences between TCR/CD3-responsive and -inert latently HIV-1 infected T cell clones, (ii) needed to be also central nodes in the network describing changes in the T cell populations of HIV/ART patients and (iii) clinically relevant inhibitors against the targets needed to be available. Based on these criteria, we chose to target (i) Src (dasatinib), (ii) Raf (sorafenib) and (iii) STAT3 (S31-201).

As the generated protein-protein interaction networks are not directional, it is not possible to predict whether e.g. an inhibitor against a central node will inhibit or activate down-stream events, in this case trigger or inhibit HIV-1 reactivation. The networks provide no information on the connection between a particular central kinase/protein, its actual role in the altered network and how this affects latency stability. Network analysis strictly increases the probability of successful target identification. We would not expect that interference with these key network nodes would directly trigger HIV-1 reactivation, but anticipated that pharmacological targeting of the selected network nodes would either boost or abrogate TCR/CD3 activation-induced HIV-1 reactivation. Ideally, interference would restore the ability of TCR/CD3 activation to trigger HIV-1 reactivation in the otherwise inert JWEAU-C6 T cells.

**Fig 10:**
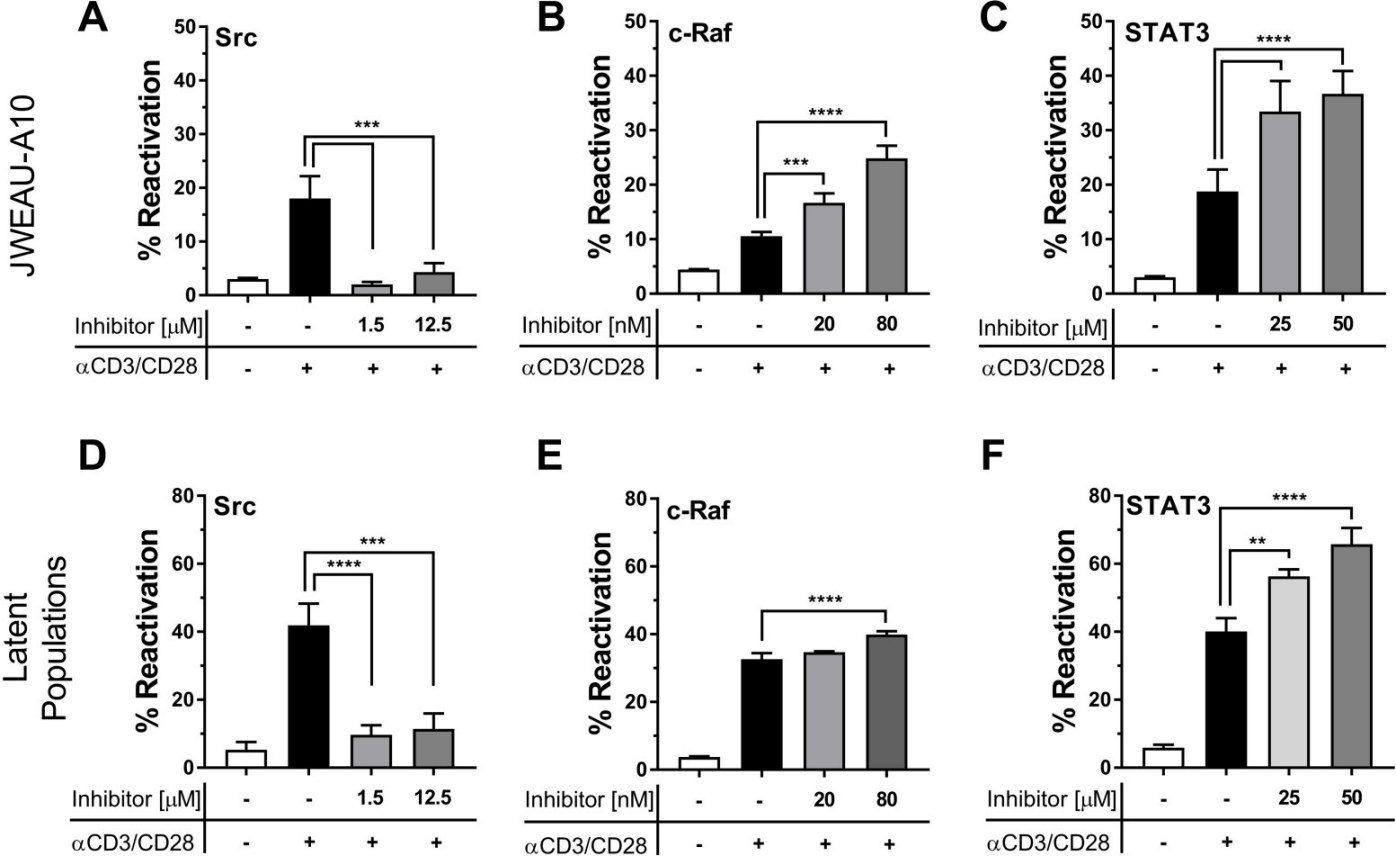
Pharmacological targeting of network hubs alters TCR/CD3 responsiveness of latently HIV-1 infected T cells. To validate that network analysis correctly predicted and prioritized drug targets that affect the ability of TCR/CD3 stimulation to trigger HIV-1 reactivation we tested the effect of inhibitors against Src, Raf and STAT3 on the ability of a-CD3/CD28 mAbs to trigger HIV-1 reactivation. JWEAU-A10 T cells (A-C) or populations enriched for latently HIV-1 infected T cells (D-F) were incubated overnight with different concentrations of the Src inhibitor dasatinib (**A, D**), the c-Raf inhibitor sorafenib (**B, E**), or the STAT3 inhibitor S31-201 (**C, F**) before stimulation with a sub-optimal concentration of α-CD3/CD28 mAbs. Data represent the mean ± standard deviation of three independent experiments.

In a first step, we pretreated JWEAU-A10 T cells or long-term HIV-1 infected T cell populations that we enriched for latently HIV-1 infected T cells (see Material and Methods section) with increasing concentrations of the three drugs, and, after 24 hours, stimulated with a suboptimal concentration of anti-CD3/CD28 mAbs. Suboptimal CD3/CD28 stimulation would facilitate the discovery of potential additive/synergistic effects of the drugs on TCR/CD3 signaling. Inhibition of Src by sub-cytotoxic concentrations of dasatinib effectively abrogated TCR/CD3 triggered HIV-1 reactivation, in both JWEAU-A10 T cells and the latently HIV-1 infected T cell population (Fig 10A and 10D) [81–84]. While this finding is likely therapeutically irrelevant, it demonstrates the ability of network software to correctly identify and prioritize functionally important protein targets. The Raf inhibitor sorafenib boosted the ability of suboptimal CD3/CD28 mAb combinations to trigger HIV-1 reactivation in JWEAU-A10 T cells in the nM-range (Fig 10B), but its effect was minimal in the latently HIV-1 infected T cell population (Fig 10E). Finally, the STAT3 inhibitor S31-201 showed robust enhancement of CD3/CD28 triggered HIV-1 reactivation in JWEAU-A10 T cells (Fig 10C), and also in the latently HIV-1 infected T cell populations (Fig 10F).

**Fig 11:**
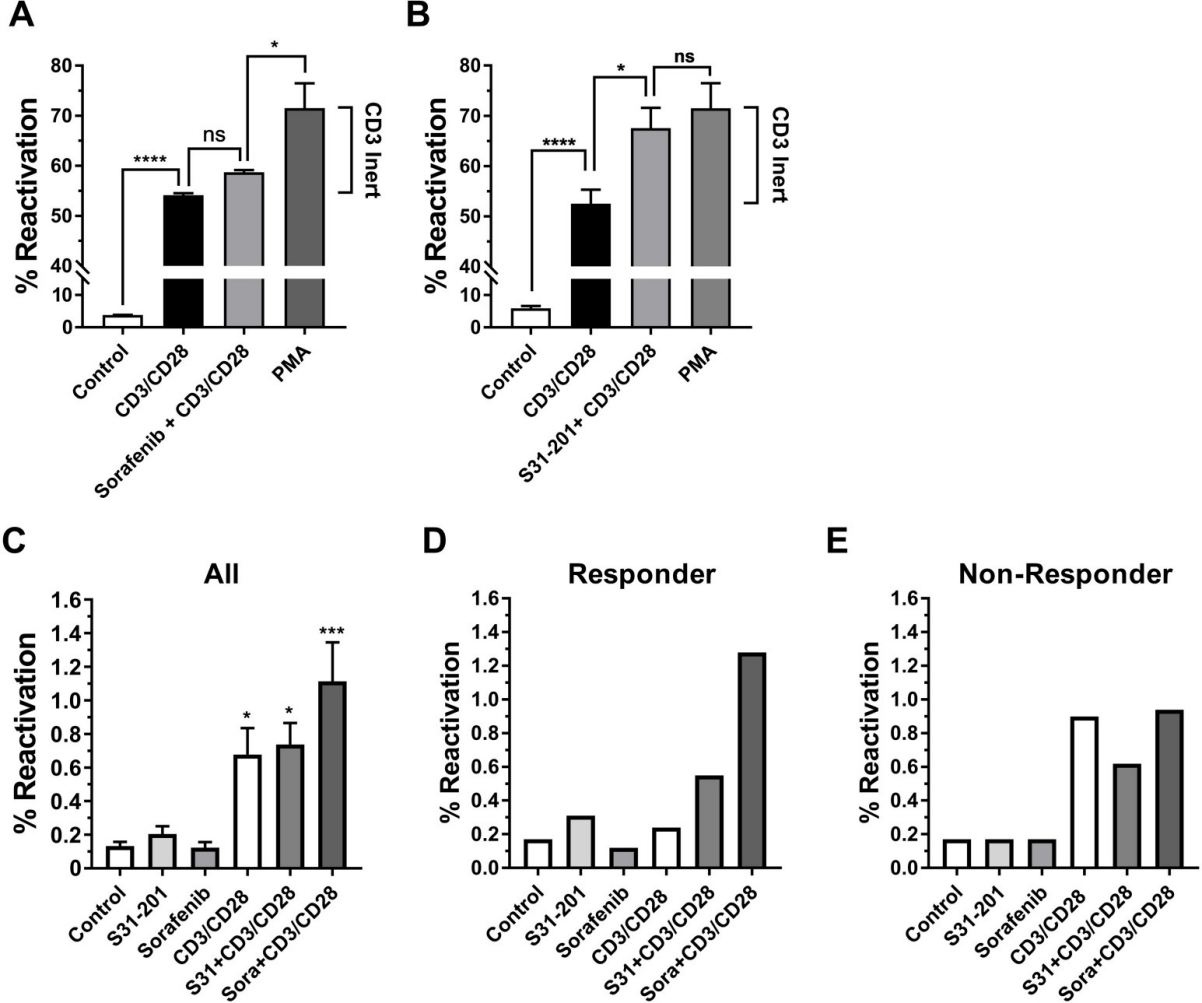
Restoring CD3 responsiveness in latently HIV-1 infected T cells. To test whether pharmacological interventions that were prioritized by network analysis would specifically restore the ability of the CD3 signaling pathway to trigger latent HIV-1 infection in CD3-inert T cells, we stimulated latently HIV-1 infected T cell populations with α-CD3/CD28 mAbs without any pretreatment or following overnight pretreatment with optimal concentrations of (**A**) the c-Raf inhibitor sorafenib or (**B**) the STAT3 inhibitor S31-201 (50 μM). PMA stimulation was used as a positive control to determine the maximum achievable reactivation level in the T cell population. The difference between the reactivation levels accomplishable by α-CD3/CD28 mAb activation and PMA stimulation constitutes the TCR/CD3-inert T cell reservoir (CD3 inert). Any increase of HIV-1 reactivation levels above the ability of α-CD3/CD28 mAbs to trigger HIV-1 reactivation means that the therapeutic intervention allows antibody stimulation to target previously activation inert latently HIV-1 infected T cells. (**C**) Using a full-length HIV-GFP reporter virus latently HIV-1 infected T cell populations were established in primary CD4+ T cell populations obtained from five different donors. The T cells were left unstimulated (control) or treated overnight with optimal concentrations of the STAT3 inhibitor S31-201 or the Raf inhibitor sorafenib. Each of these cultures was then split and left either untreated or stimulated with α-CD3/CD28 mAbs. HIV-1 reactivation was determined by flow cytometry 72 hours post α-CD3/CD28 mAbs addition using GFP expression as a surrogate marker of active HIV-1 expression. Stars indicate conditions that significantly differed from the control condition (see Statistical Analysis in methods).

We next tested whether Raf or STAT3 inhibition would permit optimal CD3/CD28 stimulation to trigger HIV-1 reactivation in the otherwise TCR/CD3 activation-inert subpopulation that is only responsive to PMA treatment. Consistent with its limited effectiveness in combination with suboptimal CD3/CD28 activation, sorafenib treatment only slightly increased the percentage of HIV-1 expressing T cells following optimal CD3/CD28 activation, but the majority of the TCR/CD3 activation-inert T cell fraction that was revealed by PMA stimulation was not addressed (Fig 11A). The STAT3 inhibitor S31-201 exhibited a stronger reactivation-boosting effect. Pretreatment with S31-201 followed by CD3/CD28 stimulation produced HIV-1 reactivation levels that were similar to the reactivation levels produced by PMA (Fig 11B), indicating that STAT3 inhibition would restore the ability of a large fraction of the initially CD3-inert T cells to generate an effective TCR/CD3 pathway signal. Of note, neither sorafenib nor S31-201 had any effect on the ability of CD3/CD28 mAb combinations to trigger HIV-1 reactivation in the inert JWEAU-C6 T cells, suggesting that extreme CD3-inertness may not be reversible (S5 Fig).

Finally, we tested whether these findings would be transferable to HIV-1 latency in primary T cells, and whether sorafenib or S31-201 would also promote reactivation of latent HIV-1 infection in an *in vitro* model of latency in primary T cell. This possibility was suggested by the similarity of signaling network changes identified in primary T cells from HIV/ART patients (Fig 8B) and latently HIV-1 infected T cell lines (Fig 9C). To test this possibility, we again generated latently HIV-1 infected primary T cell cultures using the same *in vitro* infection model utilized in Fig 1A. Similar to what was observed in latently HIV-1 infected T cell populations, neither sorafenib nor S31-201 by themselves had any direct HIV-1 reactivating effect, but both drugs exhibited the ability to boost CD3/CD28 mAb-induced HIV-1 reactivation in a donor-dependent manner. Throughout the experiments (n = 5), sorafenib provided a stronger reactivation boosting effect than S31-201, confirming that the network analysis derived target predictions could be transferred into a primary T cell-based model of latent HIV-1 infection. However, donor variation was apparent and while some infection cultures responded as predicted (Fig 11D) the drugs seem to have no boosting effect at all in other infection cultures (Fig 11E). It stands to reason that heterogeneity caused by the immunological history of primary T cells likely adds to the complexity of HIV-1 latency control in primary T cells that thus is largely, but certainly not fully reflected in latently HIV-1 infected T cell lines.

## Discussion

At the time it is assumed that latent HIV-1 infection events are established as activated T cells become HIV-1 infected before transitioning to a long-lived resting memory phenotype [1,85–89]. The resting status of memory T cells is thought to deplete HIV-1 of essential transcription factors and thereby restrict its ability to express its genes, forcing the virus into a latent state [90–92]. Based on this concept, T cell activation in general, or specifically through antibody-mediated stimulation of the TCR/CD3 complex, the experimental equivalent of cognate antigen recognition, should trigger HIV-1 reactivation. It has by now been conclusively demonstrated that this is correct only for a part of the latent HIV-1 reservoir, whereas a large portion of the latent reservoir remains recalcitrant to TCR/CD3 activation [15,16]. We thus hypothesized that a part of the HIV-1 reservoir is established in T cells that are either TCR/CD3 activation-resistant prior to infection or are rendered TCR/CD3 activation-inert by the cellular response to the actual infection event.

In this study we provide formal experimental evidence that TCR/CD3 activation-inertness (i) can indeed be generated by HIV-1 infection (Figs 1 and 2), (ii) must a fundamental mechanism that is conserved between primary T cells and T cell lines (Fig 2) and (iii) affects a substantial portion of the T cells serving as host cells for the latent HIV-1 reservoir (Figs 2 and 7). The existence of such an activation-inert reservoir T cell population would conclusively explain why cognate antigen recognition does not deplete the latent HIV-1 reservoir over time. We further demonstrate that host cells of latent HIV-1 infection events are characterized by extensive stable changes to their signaling networks (Figs 8 and 9). (iv) Our data suggest that high-level transcriptomic noise may have the potential to amplify TCR/CD3 activation-inertness (Fig 5), but (v) TCR/CD3 inertness was clearly driven by selective changes to the TCR/CD3 signaling pathway, which could be targeted and at least partially reversed by inhibitors (Figs 6 and 7).

The observed presence of stochastic gene expression changes or transcriptomic noise explains the findings by others that described a high degree of heterogeneity between individual latently HIV-1 infected T cells [44,45]. Our research extends on these studies in primary T cells and demonstrates that this diversity is not necessarily the result of a pre-existing heterogeneity caused by different immunological histories or differentiation status of T cells that later on become latently HIV-1 infected. While such T cell diversity most likely adds to the observed heterogeneity in primary T cells (Fig 11), we show that heterogeneity is generated by a fundamental mechanism that must be part of the cellular response to the actual infection event. Heterogeneity between latently HIV-1 infected T cells was observed at the gene and protein expression level (Figs 5 and 7). Given that gene expression analysis is now the basis of most studies, it was somewhat surprising that RNA-seq analysis would indicate extensive changes to the gene expression landscape, but network analysis software could not efficiently integrate these data into interaction networks or for that matter assign system-relevant motifs to the data (S1 Table). Even hierarchical clustering followed by subtree cluster analysis would not directly identify potential molecular switches that were to affect TCR/CD3 mediated HIV-1 reactivation, but rather suggested that TCR/CD3 activation impairment could be a general feature of latently HIV-1 infected T cells. This was disconcerting as the phenotype, TCR/CD3 activation-inertness, was already predetermined by functional assays (Figs 1–4). In contrast, proteomic analysis immediately identified the underlying molecular phenotype (Fig 8). The apparent failure of RNA-level analysis to identify a defined, preexisting phenotype exposes the apparent limitations of RNA-focused analysis methods. While the failure of RNA-seq analysis to detect a specified phenotype is not necessarily surprising as it is common knowledge that gene regulation effects often are not translated into protein level effects, there are no true alternatives available at the time. Proteomic analysis, no matter whether done using antibody arrays or mass spectrometry, while powerful, requires relatively large amounts of cell material. Other than transcriptomic analysis which already offers acceptable coverage of genome-wide gene expression at the single cell level, proteomic analysis is likely trailing this success by more than a decade. The inability of current proteomic analysis to perform proteome-wide analysis at the single cell level or at least with small amounts of cell material presents a major bottleneck to an immediate translation of our research approach to the study of latently infected T cells from patients or even relevant *in vitro* models of HIV-1 latency in primary T cells. Either way, the data clearly pinpoint a major shortcoming of the current RNA-level focused research efforts.

At the same time, the formal demonstration that heterogeneity at the gene expression and protein expression/phosphorylation level extends to functional diversity has implications for therapeutic strategies that seek to trigger HIV-1 reactivation in patients. The data imply that single drug interventions will be insufficient to accomplish complete viral reactivation and subsequent elimination. TCR/CD3 inertness was not the only functional consequence of the molecular changes that can occur in host cells of latent HIV-1 infected T cells. While we do not detail this phenomenon, we found that TLR pathway signaling can also be impaired (Fig 2F), which would have implications for efforts to identify HIV-1 reactivating agents that address the TLR/Myd88 pathway [33–37]. Differential reactivation responsiveness to varying stimuli, was actually paralleled by differential regulation of NF-κB dependent cellular genes, seen for the T cell activation markers CD38 and CD69 (Fig 3), and explained by the finding that while PMA would trigger an altered, but effective kinetic NF-κB signal in activation-responsive and -inert T cells, α-CD3/CD28 mAb stimulation would completely fail to trigger a NF-κB signal in the reactivation-inert JWEAU-C6 T cells, conclusively demonstrating that reactivation-inertness is indistinguishable from activation-inertness and is strictly a cellular restriction phenomenon.

This would have theoretical implication for the design of future HIV-1 cure strategies. Given that our data detail the dysfunctional nature of latently HIV-1 infected T cells, and the seemingly random nature of observed changes, it needs to be assumed that the further upstream a NF-κB activating pathway is targeted, the less likely it is that an efficient, HIV-1 reactivating NF-κB signal is being triggered. The results thus support the idea that optimal HIV-1 reactivation agents should induce NF-κB activation by targeting proteins as immediate upstream of NF-κB as possible, which reduces the likelihood that the observed stochastic changes to the host cells affect drug efficacy. Pathways that activate NF-κB, but are essential to the long-term survival of the infected T cell could be most attractive, as changes to these essential pathways would result in rapid cell death after infection. This may make the CD3 or TLR pathways suboptimal targets for LRAs, unless possibly used in a combination therapy. By the same token, the extensive heterogeneity would favor the development of drugs like SMAC mimetics such as AZD5582 that also activate the non-canonical NF-κB pathway, but more importantly, they also target an important survival pathway for cells. SMAC mimetics imitate the activity of a protein call Second Mitochondrial-derived Activator of Caspases, a pro-apoptotic mitochondrial protein that is an endogenous inhibitor of a family of cellular proteins called the Inhibitor of Apoptosis Proteins (IAPs), which in turn are important regulators of cell death and survival. The importance of such a pathway for long-term survival of the cells should make it less likely that this pathway is impaired in host cells of latent HIV-1 infection events. Functional conservation of this pathway would increase the likelihood of SMAC mimetics to broadly trigger HIV-1 reactivation within the population of latently HIV-1 infected T cells, despite the heterogeneity and pre-existing inertness in other pathways. SMAC mimetics, including AZD5582 have already been demonstrated to trigger HIV or SIV reactivation [93–95] and their potency may be increased in conjunction with drugs that restore the full signaling capacity of latently HIV-1 infected T cells.

Conceptually, the provision of experimental evidence that many host cells of latent HIV-1 infection events are extensively altered in their signaling networks and gene expression patterns and in extension, are altered in many of their functionalities, raises the possibility to affect the stability of latent HIV-1 infection events in these cells through an alternative approach. Therapeutic interventions that would restore host cell functionality may enable TCR/CD3 activation-inert latently HIV-1 infected T cells to properly respond to cognate antigen recognition, and the ensuing cellular increase in NF-κB activation would be sufficient to trigger HIV1 reactivation. Such a scenario would focus on the development of cellular reprograming strategies and remove the requirement for a potent, systemwide, stimulatory therapeutic intervention, which would here be provided by a natural process, specific antigen recognition.

## Material and methods

### Ethics statement

Human Sample disclosure and Information. Healthy, HIV-seronegative adults and HIV-seropositive subjects were recruited from the 1917 Clinic cohort at the University of Alabama at Birmingham to donate peripheral blood. All HIV-1 seropositive subjects were on ART and with undetectable viral loads (VL <50 copies/mL) for a median of 10 months (6.5–17.3 months). In accordance with the specific protocol for this project that was approved by the UAB Committee on Human Research (IRB-160715008), all subjects provided written informed consent for all biologic specimens and clinical data used in this study.

### Cell culture and reagents

All T cell lines were maintained in RPMI 1640 supplemented with 2 mM L-glutamine, 100 U/ml penicillin, 100 μg/ml streptomycin and 10% heat inactivated fetal bovine serum. Fetal bovine serum (FBS) was obtained from HyClone (Logan, Utah) and was tested on a panel of latently infected cells to assure that the utilized FBS batch did not spontaneously trigger HIV-1 reactivation [28,96]. The phorbol esters, Phorbol 12-myristate 13-acetate (PMA), prostratin, along with HMBA, and Flagellin were purchased from Sigma. Recombinant human TNF-α a well established trigger of HIV-1 reactivation was purchased from Gibco. The PKC activator Bryostatin and Sodium Butyrate (NaBu) were purchased from EMD Millipore. Vorinostat (SAHA) was purchased from Selleck chemicals. Specific Inhibitors or recombinant proteins such as the STAT3 inhibitor S31-201 (NSC 74859), the Src inhibitor dasatinib, or the c-Raf inhibitor sorafenib were all purchased from Fisher Scientific. Dasatinib is approved for the treatment of chronic myelogenous leukemia and acute lymphoblastic leukemia. Sorafenib is approved for the treatment of certain kidney and liver cancers and certain forms of Acute Myeloid Leukemia. The selective STAT3 inhibitor S31-201 had been successfully used in several mouse cancer models [97,98]. Anti-CD3/CD28 antibody-coated beads (ImmunoCult) were purchased from Stemcell technologies (Vancouver, CA).

### Model of HIV-1 latency in primary T cells

CD4+ T-cells were isolated from PBMCs from healthy donors using a negative CD4+ T cell isolation kit (Stemcell), and were then rested overnight in RPMI (10% FBS, 1% Pen/Strep, 1% L-Glutamine, and IL-2 (10 IU/ml). The T cells were stimulated with Immunocult (Stemcell) at a concentration of ~2 beads/cell and incubated at 37° for 3 days. On day three post stimulation the T cells were placed in fresh RPMI medium at a density of 5 million cells/well in a 6 well plate and infected with a HIV-GFP reporter virus (pBR43IeG-NA7nef) [99]. Infection using full-length replication competent viruses generally results in lower level reservoir formation than what can be accomplished by utilization attenuated HIV vectors that are often used by others [90,100], but fails to reproduce the impact of viral accessory genes on HIV-1 latency establishment [101]. After infection cells were pelleted by centrifugation and placed in fresh RPMI medium. After 48 hours GFP levels were determined as surrogate marker of HIV-1 infection using flow cytometry to confirm successful infection. To prevent further viral replication reverse transcriptase inhibitors 3TC(lamivudine) and EFV(efavirenz) (NIH AIDS Reagent Program) were added and the cells were cultured for 30 days. Medium was replenished twice a week. After 30 days the T cell cultures were sorted using a BD FACSAria cell sorter to remove all remaining GFP+, and therefore actively infected T cells to reduce the signal background. Cells were then plated at a density of 5x10^5^ to 1x10^6^/ml into the described experimental conditions. 3 days post reactivation the cell cultures were analyzed for the frequency of GFP+ T cells using a BD LSRII flow cytometer acquiring at least 100,000 events per experimental condition.

### Generation of latently HIV-1 infected T cell populations and T cell clones

The latently HIV-1 infected T cell populations were generated by infecting a Jurkat T cell-based reporter cell population (JR5D4 cells) [30] with the primary HIV-1 patient isolate WEAU [32] or the classic laboratory strain HIV-1 NL43. Starting two days post infection (3TC) was continuously added to prevent viral replication and the establishment of pre-integration latency. On day 4 post infection we selected cultures with infection levels around 40% were selected and maintained until day ~60 when active infection levels had mostly declined to levels between 1–5%. In the HIV-1 WEAU infected T cell population, addition of PMA revealed the presence of a reactivatable HIV-1 reservoir that varied in size between 4–10%. The HIV-1 WEAU infected bulk cell populations were plated in a limiting dilution and tested for CD3 inertness and PMA responsiveness, to generate the differentially responsive cell clones JWEAU-A10 (responsive) and JWEAU-C6 (inert). To generate T cell populations that were enriched for latently HIV-1 infected T cells, we exploited the reported ability of host cells of latent HIV-1 infection events to repeatedly shut down the virus into a latent state. HIV-1 reactivation was triggered using PMA and on day 2 post activation cell sorting was performed for GFP+ T cells. Over a period of 3–4 weeks, active HIV-1 infection in these cells was again shut down into a latent state, resulting in an enriched population in which ~80% of the cells would hold reactivatable, latent HIV-1 infection events.

### TransAM assays to resolve kinetic NF-κB response

T cells were stimulated with either PMA or anti-CD3/CD28 mAb combinations and cell material was harvested at different time points post stimulation (10x10e6 cells per time point). Nuclear extracts were generated and NF-κB p65 activity in the nuclear extracts of cells was determined using TransAM assays (Active Motif). All experiments were performed according to the manufacturer's instructions. TransAM assays measure the ability of activated NF-κB to bind to an NF-κB consensus sequence in solution, with a 5- to 10-fold-higher sensitivity than gel shift assays. At 0 and 24hrs post stimulation one million cells were harvested for activation marker staining, and compared to isotype controls.

### Kinexus antibody microarray-based analysis

Kinome analysis to study protein expression and phosphorylation levels was done using Kinexus microarray analysis. 50 μg of cell lysate protein (~5x10^6^ cells) from each sample or experimental condition were covalently labeled with a proprietary fluorescent dye according to the manufacturer’s instructions (Kinexus, Canada). After the completion of the labeling reaction, any free dye was removed by gel filtration. After blocking non-specific binding sites on the array, an incubation chamber was mounted onto the microarray to permit the loading of two side by side samples on the same chip. Following sample incubation, unbound proteins were washed away. KAM-850 arrays detect 189 protein kinases, 31 protein phosphatases and 142 regulatory subunits of these enzymes and other cell signaling proteins. This array provided information on the phosphorylation state of 128 unique sites in protein kinases, 4 sites in protein phosphatases and 155 sites in other cell signaling proteins. KAM-900 chips are spotted in duplicates with over 870 antibodies. 265 pan-specific antibodies and 613 phosphosite specific antibodies. Each array produced a pair of 16-bit images, which are captured with a Perkin-Elmer ScanArray Reader laser array scanner (Waltham, MA). Signal quantification was performed with ImaGene 8.0 from BioDiscovery (El Segundo, CA) with predetermined settings for spot segmentation and background correction. The background-corrected raw intensity data were logarithmically transformed with base 2. Since Z normalization in general displays greater stability as a result of examining where each signal falls in the overall distribution of values within a given sample, as opposed to adjusting all of the signals in a sample by a single common value, Z scores are calculated by subtracting the overall average intensity of all spots within a sample from the raw intensity for each spot, and dividing it by the standard deviations (SD) of all of the measured intensities within each sample [102]. Z’ ratios were further calculated by taking the difference between the averages of the observed protein Z scores and dividing by the SD of all of the differences for that particular comparison. Calculated Z’ ratios have the advantage that they can be used in multiple comparisons without further reference to the individual conditional standard deviations by which they were derived.

### RNA-seq analysis

Total RNA was extracted using the Qiagen RNeasy mini kit. Genewiz (Plainfield, NJ) prepared cDNA libraries and performed sequencing. For RNA-seq data processing and analysis, raw paired reads were first adapter-trimmed from fastq files using TrimGalore! (http://www.bioinformatics.babraham. ac.uk/projects/trim_galore/). Reads were aligned to Hg19 using STAR [103], and count matrices were generated using HTSeq-count [104]. DESeq2 was used to normalize read counts (CPM) and analyze differential expression [105]. For the analysis of global transcriptional regulation, genes were considered differentially expressed if they had an adjusted *P*-value <0.01 by the likelihood ratio test. For pairwise comparisons, genes were considered differentially expressed if they had at least a 1.5-fold change, adjusted *P*-value < 0.01, and at least one signal > 250 CPM, as qPCR validation repeatedly failed to confirm lower signals. Pheatmap (*) was used to row-normalize, cluster (via hclust, using the complete linkage method), and visualize global transcriptional changes. Gene Ontology analysis was conducted using Metascape [43].

### Network analysis

MetaCore software (Clarivate Analytics) was used to generate shortest pathway interaction networks that are presented. Pathway specific GO filters or tissue specific filters were used to prioritize nodes and edges. MetaCore was further used to identify pathway associations of the identified input dataset, using network enrichment analysis.

### Statistical analysis

Statistical significance of differences between multiple experimental conditions was determined by ANOVA with multiple comparisons (Tukey’s correction) (*p<0.05, **p<0.01, ***p<0.001, ****p<0.0001) or students T-test for comparison between two conditions.

## Supporting information

S1 Fig Representative flow cytometric dot plot analysis of GFP expression as a surrogate marker of HIV-1 expression in the TCR/CD3-responsive JWEAU-A10 T cells and the TCR/CD3-inert JWEAU-C6 T cells at baseline (control) and following activation by α-CD3/CD28 mAb or PMA. (TIF)

S2 Fig Motif enrichment analysis of the genes in each cluster of the RNA-seq analysis data shown in Fig 5 was performed using MetaScape [43]. The top 5-ranked motifs for each motif are listed and the p-values for each motif are depicted as histograms.
(TIF)

S3 Fig Focused analysis of the genes involved in T cell activation (GO term 0042110) from the RNA-seq data set presented in Fig 5 visualizes the presence of extensive difference between uninfected and latently HIV-1 infected T cells, while demonstrating the absence of relevant differences between the latently HIV-1 infected T cell clones in this pathway motif. (TIF)

S4 Fig Protein-protein interaction network for the RNA-seq data describing the shared transcriptomic features between activation-inert and activation-responsive latently HIV-1 infected T cells that differ from uninfected T cells. (**A**) The depiction visualizes the lack of connectivity of a large portion of seed nodes (proteins) around a central interaction network. (**B**) Visualization of the core network hiding the unconnected genes.
(TIF)

S5 Fig Targeting network hubs fails to restore reactivation responsiveness to TCR/CD3 stimulation in CD3-activation inert latently HIV-1 infected T cells. To determine if Src, Raf and STAT3 inhibition would also affect or restore the TCR/CD3 responsiveness of JWEAU-C6 T cells, dasatinib (Src), sorafenib (Raf) or S31-201 (STAT3) were titrated on JWEAU-C6 T cells, which were then stimulated with αCD3/CD28 mAbs. HIV-1 reactivation was determined after 24h by flow cytometric analysis using GFP expression as a surrogate marker of active HIV-1 infection. Data represent the mean ± standard deviation of three independent experiments.
(TIF)

S1 Table Network enrichment analysis of genes that based on RNA-seq analysis data are differentially expressed in the activation -responsive JWEAU-A10 T cells than in the activation-inert JWEAU-C6 T cells. (TIF)

## References

[1] Finzi D, Blankson J, Siliciano JD, Margolick JB, Chadwick K, Pierson T, et al Latent infection of CD4+ T cells provides a mechanism for lifelong persistence of HIV-1, even in patients on effective combination therapy. Nat Med. 1999;5(5):512–7. 10.1038/8394 . 10229227

[2] Chun TW, Carruth L, Finzi D, Shen X, DiGiuseppe JA, Taylor H, et al Quantification of latent tissue reservoirs and total body viral load in HIV-1 infection. Nature. 1997;387(6629):183–8. 10.1038/387183a0 . 9144289

[3] Chun TW, Davey RT Jr, Ostrowski M, Shawn Justement J, Engel D, Mullins JI, et al Relationship between pre-existing viral reservoirs and the re-emergence of plasma viremia after discontinuation of highly active anti-retroviral therapy. Nat Med. 2000;6(7):757–61. Epub 2000/07/11. 10.1038/77481 . 10888923

[4] Chun TW, Engel D, Mizell SB, Ehler LA, Fauci AS. Induction of HIV-1 replication in latently infected CD4+ T cells using a combination of cytokines. J Exp Med. 1998;188(1):83–91. 10.1084/jem.188.1.83 . 9653086 PMC2525548

[5] Macallan DC, Wallace D, Zhang Y, De Lara C, Worth AT, Ghattas H, et al Rapid turnover of effector-memory CD4(+) T cells in healthy humans. J Exp Med. 2004;200(2):255–60. Epub 2004/07/14. 10.1084/jem.20040341 15249595 PMC2212011

[6] Vrisekoop N, den Braber I, de Boer AB, Ruiter AF, Ackermans MT, van der Crabben SN, et al Sparse production but preferential incorporation of recently produced naive T cells in the human peripheral pool. Proc Natl Acad Sci U S A. 2008;105(16):6115–20. Epub 2008/04/19. 10.1073/pnas.0709713105 18420820 PMC2329696

[7] Hellerstein M, Hanley MB, Cesar D, Siler S, Papageorgopoulos C, Wieder E, et al Directly measured kinetics of circulating T lymphocytes in normal and HIV-1-infected humans. Nat Med. 1999;5(1):83–9. Epub 1999/01/12. 10.1038/4772 . 9883844

[8] Ladell K, Hellerstein MK, Cesar D, Busch R, Boban D, McCune JM. Central memory CD8+ T cells appear to have a shorter lifespan and reduced abundance as a function of HIV disease progression. J Immunol. 2008;180(12):7907–18. Epub 2008/06/05. 10.4049/jimmunol.180.12.7907 18523254 PMC2562545

[9] Bosque A, Famiglietti M, Weyrich A, Goulston C, Planelles V. Homeostatic Proliferation Fails to Efficiently Reactivate HIV-1 Latently Infected Central Memory CD4+ T Cells. PLoS Pathog. 2011;In Press. 10.1371/journal.ppat.1002288 21998586 PMC3188522

[10] Maldarelli F, Wu X, Su L, Simonetti FR, Shao W, Hill S, et al HIV latency. Specific HIV integration sites are linked to clonal expansion and persistence of infected cells. Science. 2014;345(6193):179–83. Epub 2014/06/28. 10.1126/science.1254194 24968937 PMC4262401

[11] Wange RL. LAT, the linker for activation of T cells: a bridge between T cell-specific and general signaling pathways. Sci STKE. 2000;2000(63):re1 Epub 2001/12/26. 10.1126/stke.2000.63.re1 . 11752630

[12] Fraser C, Ferguson NM, Ghani AC, Prins JM, Lange JM, Goudsmit J, et al Reduction of the HIV-1-infected T-cell reservoir by immune activation treatment is dose-dependent and restricted by the potency of antiretroviral drugs. Aids. 2000;14(6):659–69. 10.1097/00002030-200004140-00005 . 10807189

[13] Kulkosky J, Nunnari G, Otero M, Calarota S, Dornadula G, Zhang H, et al Intensification and stimulation therapy for human immunodeficiency virus type 1 reservoirs in infected persons receiving virally suppressive highly active antiretroviral therapy. J Infect Dis. 2002;186(10):1403–11. 10.1086/344357 12404155

[14] van Praag RM, Prins JM, Roos MT, Schellekens PT, Ten Berge IJ, Yong SL, et al OKT3 and IL-2 treatment for purging of the latent HIV-1 reservoir in vivo results in selective long-lasting CD4+ T cell depletion. J Clin Immunol. 2001;21(3):218–26. Epub 2001/06/14. 10.1023/a:1011091300321 . 11403229

[15] Ho YC, Shan L, Hosmane NN, Wang J, Laskey SB, Rosenbloom DI, et al Replication-competent noninduced proviruses in the latent reservoir increase barrier to HIV-1 cure. Cell. 2013;155(3):540–51. Epub 2013/11/19. 10.1016/j.cell.2013.09.020 24243014 PMC3896327

[16] Rezaei SD, Lu HK, Chang JJ, Rhodes A, Lewin SR, Cameron PU. The Pathway To Establishing HIV Latency Is Critical to How Latency Is Maintained and Reversed. J Virol. 2018;92(13). Epub 2018/04/13. 10.1128/JVI.02225-17 29643247 PMC6002734

[17] Shishido T, Wolschendorf F, Duverger A, Wagner F, Kappes J, Jones J, et al Selected Drugs with Reported Secondary Cell-Differentiating Capacity Prime Latent HIV-1 Infection for Reactivation. J Virol. 2012;86(17):9055–69. Epub 2012/06/15. 10.1128/JVI.00793-12 22696646 PMC3416156

[18] Wolschendorf F, Bosque A, Shishido T, Duverger A, Jones J, Planelles V, et al Kinase control prevents HIV-1 reactivation in spite of high levels of induced NF-kappaB activity. J Virol. 2012 Epub 2012/02/22. 10.1128/JVI.06726-11 . 22345467 PMC3318643

[19] Duverger A, Wolschendorf F, Anderson JC, Wagner F, Bosque A, Shishido T, et al Kinase control of latent HIV-1 infection: PIM-1 kinase as a major contributor to HIV-1 reactivation. J Virol. 2014;88(1):364–76. Epub 2013/10/25. 10.1128/JVI.02682-13 24155393 PMC3911731

[20] Bosque A, Planelles V. Studies of HIV-1 latency in an ex vivo model that uses primary central memory T cells. Methods. 53(1):54–61. Epub 2010/10/26. S1046-2023(10)00252-5 [pii] 10.1016/j.ymeth.2010.10.002 20970502 PMC3031099

[21] Bosque A, Planelles V. Induction of HIV-1 latency and reactivation in primary memory CD4+ T cells. Blood. 2009;113(1):58–65. Epub 2008/10/14. blood-2008-07-168393 [pii] 10.1182/blood-2008-07-168393 18849485 PMC2614643

[22] Cameron PU, Saleh S, Sallmann G, Solomon A, Wightman F, Evans VA, et al Establishment of HIV-1 latency in resting CD4+ T cells depends on chemokine-induced changes in the actin cytoskeleton. Proc Natl Acad Sci U S A. 2010;107(39):16934–9. Epub 2010/09/15. 10.1073/pnas.1002894107 20837531 PMC2947912

[23] Kim JE, White FM. Quantitative analysis of phosphotyrosine signaling networks triggered by CD3 and CD28 costimulation in Jurkat cells. J Immunol. 2006;176(5):2833–43. Epub 2006/02/24. 10.4049/jimmunol.176.5.2833 . 16493040

[24] Tong-Starkesen SE, Luciw PA, Peterlin BM. Signaling through T lymphocyte surface proteins, TCR/CD3 and CD28, activates the HIV-1 long terminal repeat. J Immunol. 1989;142(2):702–7. Epub 1989/01/15. . 2536062

[25] Manger B, Weiss A, Imboden J, Laing T, Stobo JD. The role of protein kinase C in transmembrane signaling by the T cell antigen receptor complex. Effects of stimulation with soluble or immobilized CD3 antibodies. J Immunol. 1987;139(8):2755–60. Epub 1987/10/15. . 3116093

[26] Ledbetter JA, Gentry LE, June CH, Rabinovitch PS, Purchio AF. Stimulation of T cells through the CD3/T-cell receptor complex: role of cytoplasmic calcium, protein kinase C translocation, and phosphorylation of pp60c-src in the activation pathway. Mol Cell Biol. 1987;7(2):650–6. Epub 1987/02/01. 10.1128/mcb.7.2.650 2434833 PMC365120

[27] Jordan A, Bisgrove D, Verdin E. HIV reproducibly establishes a latent infection after acute infection of T cells in vitro. Embo J. 2003;22(8):1868–77. 10.1093/emboj/cdg188 . 12682019 PMC154479

[28] Kutsch O, Benveniste EN, Shaw GM, Levy DN. Direct and quantitative single-cell analysis of human immunodeficiency virus type 1 reactivation from latency. J Virol. 2002;76(17):8776–86. 10.1128/jvi.76.17.8776-8786.2002 12163598 PMC136999

[29] Duverger A, Jones J, May J, Bibollet-Ruche F, Wagner FA, Cron RQ, et al Determinants of the establishment of human immunodeficiency virus type 1 latency. J Virol. 2009;83(7):3078–93. Epub 2009/01/16. JVI.02058-08 [pii] 10.1128/JVI.02058-08 19144703 PMC2655589

[30] Duverger A, Wolschendorf F, Zhang M, Wagner F, Hatcher B, Jones J, et al An AP-1 Binding Site in the Enhancer/Core Element of the HIV-1 Promoter Controls the Ability of HIV-1 To Establish Latent Infection. J Virol. 2013;87(4):2264–77. Epub 2012/12/14. 10.1128/JVI.01594-12 . 23236059 PMC3571467

[31] Seu L, Sabbaj S, Duverger A, Wagner F, Anderson JC, Davies E, et al Stable Phenotypic Changes of the Host T Cells Are Essential to the Long-Term Stability of Latent HIV-1 Infection. J Virol. 2015;89(13):6656–72. Epub 2015/04/17. 10.1128/JVI.00571-15 25878110 PMC4468477

[32] Wei X, Decker JM, Wang S, Hui H, Kappes JC, Wu X, et al Antibody neutralization and escape by HIV-1. Nature. 2003;422(6929):307–12. Epub 2003/03/21. 10.1038/nature01470 [pii]. . 12646921

[33] Novis CL, Archin NM, Buzon MJ, Verdin E, Round JL, Lichterfeld M, et al Reactivation of latent HIV-1 in central memory CD4(+) T cells through TLR-1/2 stimulation. Retrovirology. 2013;10:119 Epub 2013/10/26. 10.1186/1742-4690-10-119 24156240 PMC3826617

[34] Tsai A, Irrinki A, Kaur J, Cihlar T, Kukolj G, Sloan DD, et al Toll-Like Receptor 7 Agonist GS-9620 Induces HIV Expression and HIV-Specific Immunity in Cells from HIV-Infected Individuals on Suppressive Antiretroviral Therapy. J Virol. 2017;91(8). Epub 2017/02/10. 10.1128/JVI.02166-16 28179531 PMC5375698

[35] Meas HZ, Haug M, Beckwith MS, Louet C, Ryan L, Hu Z, et al Sensing of HIV-1 by TLR8 activates human T cells and reverses latency. Nat Commun. 2020;11(1):147 Epub 2020/01/11. 10.1038/s41467-019-13837-4 31919342 PMC6952430

[36] Macedo AB, Novis CL, Bosque A. Targeting Cellular and Tissue HIV Reservoirs With Toll-Like Receptor Agonists. Front Immunol. 2019;10:2450 Epub 2019/11/05. 10.3389/fimmu.2019.02450 31681325 PMC6804373

[37] Lim SY, Osuna CE, Hraber PT, Hesselgesser J, Gerold JM, Barnes TL, et al TLR7 agonists induce transient viremia and reduce the viral reservoir in SIV-infected rhesus macaques on antiretroviral therapy. Sci Transl Med. 2018;10(439). Epub 2018/05/04. 10.1126/scitranslmed.aao4521 29720451 PMC5973480

[38] Shubinsky G, Schlesinger M. The CD38 lymphocyte differentiation marker: new insight into its ectoenzymatic activity and its role as a signal transducer. Immunity. 1997;7(3):315–24. Epub 1997/11/05. 10.1016/s1074-7613(00)80353-2 . 9324352

[39] Lopez-Cabrera M, Santis AG, Fernandez-Ruiz E, Blacher R, Esch F, Sanchez-Mateos P, et al Molecular cloning, expression, and chromosomal localization of the human earliest lymphocyte activation antigen AIM/CD69, a new member of the C-type animal lectin superfamily of signal-transmitting receptors. J Exp Med. 1993;178(2):537–47. Epub 1993/08/01. 10.1084/jem.178.2.537 8340758 PMC2191117

[40] Cosulich ME, Rubartelli A, Risso A, Cozzolino F, Bargellesi A. Functional characterization of an antigen involved in an early step of T-cell activation. Proc Natl Acad Sci U S A. 1987;84(12):4205–9. Epub 1987/06/01. 10.1073/pnas.84.12.4205 3295878 PMC305053

[41] Castellanos MC, Munoz C, Montoya MC, Lara-Pezzi E, Lopez-Cabrera M, de Landazuri MO. Expression of the leukocyte early activation antigen CD69 is regulated by the transcription factor AP-1. J Immunol. 1997;159(11):5463–73. Epub 1998/05/15. . 9580241

[42] Hoffmann A, Levchenko A, Scott ML, Baltimore D. The IkappaB-NF-kappaB signaling module: temporal control and selective gene activation. Science. 2002;298(5596):1241–5. Epub 2002/11/09. 10.1126/science.1071914 . 12424381

[43] Zhou Y, Zhou B, Pache L, Chang M, Khodabakhshi AH, Tanaseichuk O, et al Metascape provides a biologist-oriented resource for the analysis of systems-level datasets. Nat Commun. 2019;10(1):1523 Epub 2019/04/05. 10.1038/s41467-019-09234-6 30944313 PMC6447622

[44] Bradley T, Ferrari G, Haynes BF, Margolis DM, Browne EP. Single-Cell Analysis of Quiescent HIV Infection Reveals Host Transcriptional Profiles that Regulate Proviral Latency. Cell Rep. 2018;25(1):107–17 e3. Epub 2018/10/04. 10.1016/j.celrep.2018.09.020 30282021 PMC6258175

[45] Golumbeanu M, Cristinelli S, Rato S, Munoz M, Cavassini M, Beerenwinkel N, et al Single-Cell RNA-Seq Reveals Transcriptional Heterogeneity in Latent and Reactivated HIV-Infected Cells. Cell Rep. 2018;23(4):942–50. Epub 2018/04/26. 10.1016/j.celrep.2018.03.102 . 29694901

[46] Gryder BE, Wu L, Woldemichael GM, Pomella S, Quinn TR, Park PMC, et al Chemical genomics reveals histone deacetylases are required for core regulatory transcription. Nat Commun. 2019;10(1):3004 Epub 2019/07/10. 10.1038/s41467-019-11046-7 31285436 PMC6614369

[47] Beliakova-Bethell N, Zhang JX, Singhania A, Lee V, Terry VH, Richman DD, et al Suberoylanilide hydroxamic acid induces limited changes in the transcriptome of primary CD4(+) T cells. AIDS. 2013;27(1):29–37. Epub 2012/12/12. 10.1097/QAD.0b013e32835b3e26 23221426 PMC3752851

[48] LaBonte MJ, Wilson PM, Fazzone W, Groshen S, Lenz HJ, Ladner RD. DNA microarray profiling of genes differentially regulated by the histone deacetylase inhibitors vorinostat and LBH589 in colon cancer cell lines. BMC Med Genomics. 2009;2:67 Epub 2009/12/02. 10.1186/1755-8794-2-67 19948057 PMC2799439

[49] Glaser KB, Staver MJ, Waring JF, Stender J, Ulrich RG, Davidsen SK. Gene expression profiling of multiple histone deacetylase (HDAC) inhibitors: defining a common gene set produced by HDAC inhibition in T24 and MDA carcinoma cell lines. Mol Cancer Ther. 2003;2(2):151–63. Epub 2003/02/18. . 12589032

[50] Dawson MA, Prinjha RK, Dittmann A, Giotopoulos G, Bantscheff M, Chan WI, et al Inhibition of BET recruitment to chromatin as an effective treatment for MLL-fusion leukaemia. Nature. 2011;478(7370):529–33. Epub 2011/10/04. 10.1038/nature10509 21964340 PMC3679520

[51] LeRoy G, Rickards B, Flint SJ. The double bromodomain proteins Brd2 and Brd3 couple histone acetylation to transcription. Mol Cell. 2008;30(1):51–60. Epub 2008/04/15. 10.1016/j.molcel.2008.01.018 18406326 PMC2387119

[52] Wu SY, Chiang CM. The double bromodomain-containing chromatin adaptor Brd4 and transcriptional regulation. J Biol Chem. 2007;282(18):13141–5. Epub 2007/03/03. 10.1074/jbc.R700001200 . 17329240

[53] Patel MC, Debrosse M, Smith M, Dey A, Huynh W, Sarai N, et al BRD4 coordinates recruitment of pause release factor P-TEFb and the pausing complex NELF/DSIF to regulate transcription elongation of interferon-stimulated genes. Mol Cell Biol. 2013;33(12):2497–507. Epub 2013/04/17. 10.1128/MCB.01180-12 23589332 PMC3700095

[54] Bartholomeeusen K, Xiang Y, Fujinaga K, Peterlin BM. Bromodomain and extra-terminal (BET) bromodomain inhibition activate transcription via transient release of positive transcription elongation factor b (P-TEFb) from 7SK small nuclear ribonucleoprotein. J Biol Chem. 2012;287(43):36609–16. Epub 2012/09/07. 10.1074/jbc.M112.410746 22952229 PMC3476326

[55] Yang Z, Yik JH, Chen R, He N, Jang MK, Ozato K, et al Recruitment of P-TEFb for stimulation of transcriptional elongation by the bromodomain protein Brd4. Mol Cell. 2005;19(4):535–45. Epub 2005/08/20. 10.1016/j.molcel.2005.06.029 . 16109377

[56] Jang MK, Mochizuki K, Zhou M, Jeong HS, Brady JN, Ozato K. The bromodomain protein Brd4 is a positive regulatory component of P-TEFb and stimulates RNA polymerase II-dependent transcription. Mol Cell. 2005;19(4):523–34. Epub 2005/08/20. 10.1016/j.molcel.2005.06.027 . 16109376

[57] Dar RD, Hosmane NN, Arkin MR, Siliciano RF, Weinberger LS. Screening for noise in gene expression identifies drug synergies. Science. 2014;344(6190):1392–6. Epub 2014/06/07. 10.1126/science.1250220 24903562 PMC4122234

[58] Beliakova-Bethell N, Mukim A, White CH, Deshmukh S, Abewe H, Richman DD, et al Histone deacetylase inhibitors induce complex host responses that contribute to differential potencies of these compounds in HIV reactivation. J Biol Chem. 2019;294(14):5576–89. Epub 2019/02/13. 10.1074/jbc.RA118.005185 30745362 PMC6462528

[59] Vlach J, Pitha PM. Hexamethylene bisacetamide activates the human immunodeficiency virus type 1 provirus by an NF-kappa B-independent mechanism. J Gen Virol. 1993;74 (Pt 11):2401–8. Epub 1993/11/01. 10.1099/0022-1317-74-11-2401 . 8245855

[60] Contreras X, Barboric M, Lenasi T, Peterlin BM. HMBA releases P-TEFb from HEXIM1 and 7SK snRNA via PI3K/Akt and activates HIV transcription. PLoS Pathog. 2007;3(10):1459–69. Epub 2007/10/17. 07-PLPA-RA-0377 [pii] 10.1371/journal.ppat.0030146 17937499 PMC2014796

[61] Boehm D, Calvanese V, Dar RD, Xing S, Schroeder S, Martins L, et al BET bromodomain-targeting compounds reactivate HIV from latency via a Tat-independent mechanism. Cell Cycle. 2013;12(3):452–62. 10.4161/cc.23309 23255218 PMC3587446

[62] Li Z, Guo J, Wu Y, Zhou Q. The BET bromodomain inhibitor JQ1 activates HIV latency through antagonizing Brd4 inhibition of Tat-transactivation. Nucleic Acids Res. 2013;41(1):277–87. 10.1093/nar/gks976 23087374 PMC3592394

[63] Zhu J, Gaiha GD, John SP, Pertel T, Chin CR, Gao G, et al Reactivation of latent HIV-1 by inhibition of BRD4. Cell Rep. 2012;2(4):807–16. 10.1016/j.celrep.2012.09.008 23041316 PMC3523124

[64] Gaiha GD, McKim KJ, Woods M, Pertel T, Rohrbach J, Barteneva N, et al Dysfunctional HIV-specific CD8+ T cell proliferation is associated with increased caspase-8 activity and mediated by necroptosis. Immunity. 2014;41(6):1001–12. Epub 2014/12/20. 10.1016/j.immuni.2014.12.011 25526311 PMC4312487

[65] Yamamoto T, Price DA, Casazza JP, Ferrari G, Nason M, Chattopadhyay PK, et al Surface expression patterns of negative regulatory molecules identify determinants of virus-specific CD8+ T-cell exhaustion in HIV infection. Blood. 2011;117(18):4805–15. Epub 2011/03/15. 10.1182/blood-2010-11-317297 21398582 PMC3100691

[66] Quigley M, Pereyra F, Nilsson B, Porichis F, Fonseca C, Eichbaum Q, et al Transcriptional analysis of HIV-specific CD8+ T cells shows that PD-1 inhibits T cell function by upregulating BATF. Nat Med. 2010;16(10):1147–51. Epub 2010/10/05. 10.1038/nm.2232 20890291 PMC3326577

[67] Migueles SA, Osborne CM, Royce C, Compton AA, Joshi RP, Weeks KA, et al Lytic granule loading of CD8+ T cells is required for HIV-infected cell elimination associated with immune control. Immunity. 2008;29(6):1009–21. Epub 2008/12/09. 10.1016/j.immuni.2008.10.010 19062316 PMC2622434

[68] Wherry EJ, Ha SJ, Kaech SM, Haining WN, Sarkar S, Kalia V, et al Molecular signature of CD8+ T cell exhaustion during chronic viral infection. Immunity. 2007;27(4):670–84. Epub 2007/10/24. 10.1016/j.immuni.2007.09.006 . 17950003

[69] Day CL, Kiepiela P, Leslie AJ, van der Stok M, Nair K, Ismail N, et al Proliferative capacity of epitope-specific CD8 T-cell responses is inversely related to viral load in chronic human immunodeficiency virus type 1 infection. J Virol. 2007;81(1):434–8. Epub 2006/10/20. 10.1128/JVI.01754-06 17050606 PMC1797250

[70] Day CL, Kaufmann DE, Kiepiela P, Brown JA, Moodley ES, Reddy S, et al PD-1 expression on HIV-specific T cells is associated with T-cell exhaustion and disease progression. Nature. 2006;443(7109):350–4. Epub 2006/08/22. 10.1038/nature05115 . 16921384

[71] Trautmann L, Janbazian L, Chomont N, Said EA, Gimmig S, Bessette B, et al Upregulation of PD-1 expression on HIV-specific CD8+ T cells leads to reversible immune dysfunction. Nat Med. 2006;12(10):1198–202. Epub 2006/08/19. 10.1038/nm1482 . 16917489

[72] Migueles SA, Laborico AC, Shupert WL, Sabbaghian MS, Rabin R, Hallahan CW, et al HIV-specific CD8+ T cell proliferation is coupled to perforin expression and is maintained in nonprogressors. Nat Immunol. 2002;3(11):1061–8. Epub 2002/10/09. 10.1038/ni845 . 12368910

[73] Driessens G, Zheng Y, Locke F, Cannon JL, Gounari F, Gajewski TF. Beta-catenin inhibits T cell activation by selective interference with linker for activation of T cells-phospholipase C-gamma1 phosphorylation. J Immunol. 2011;186(2):784–90. Epub 2010/12/15. 10.4049/jimmunol.1001562 21149602 PMC4888792

[74] Yu Q, Sharma A, Sen JM. TCF1 and beta-catenin regulate T cell development and function. Immunol Res. 2010;47(1–3):45–55. Epub 2010/01/19. 10.1007/s12026-009-8137-2 20082155 PMC2891409

[75] Xu Y, Banerjee D, Huelsken J, Birchmeier W, Sen JM. Deletion of beta-catenin impairs T cell development. Nat Immunol. 2003;4(12):1177–82. Epub 2003/11/11. 10.1038/ni1008 . 14608382

[76] Gounari F, Aifantis I, Khazaie K, Hoeflinger S, Harada N, Taketo MM, et al Somatic activation of beta-catenin bypasses pre-TCR signaling and TCR selection in thymocyte development. Nat Immunol. 2001;2(9):863–9. Epub 2001/08/30. 10.1038/ni0901-863 . 11526403

[77] Ioannidis V, Beermann F, Clevers H, Held W. The beta-catenin—TCF-1 pathway ensures CD4(+)CD8(+) thymocyte survival. Nat Immunol. 2001;2(8):691–7. Epub 2001/07/31. 10.1038/90623 . 11477404

[78] Hui E, Vale RD. In vitro membrane reconstitution of the T-cell receptor proximal signaling network. Nat Struct Mol Biol. 2014;21(2):133–42. Epub 2014/01/28. 10.1038/nsmb.2762 24463463 PMC4062301

[79] Cloutier JF, Veillette A. Cooperative inhibition of T-cell antigen receptor signaling by a complex between a kinase and a phosphatase. J Exp Med. 1999;189(1):111–21. Epub 1999/01/05. 10.1084/jem.189.1.111 9874568 PMC1887684

[80] Takeuchi M, Kuramochi S, Fusaki N, Nada S, Kawamura-Tsuzuku J, Matsuda S, et al Functional and physical interaction of protein-tyrosine kinases Fyn and Csk in the T-cell signaling system. J Biol Chem. 1993;268(36):27413–9. Epub 1993/12/25. . 8262983

[81] Mestermann K, Giavridis T, Weber J, Rydzek J, Frenz S, Nerreter T, et al The tyrosine kinase inhibitor dasatinib acts as a pharmacologic on/off switch for CAR T cells. Sci Transl Med. 2019;11(499). Epub 2019/07/05. 10.1126/scitranslmed.aau5907 . 31270272 PMC7523030

[82] Lee KC, Ouwehand I, Giannini AL, Thomas NS, Dibb NJ, Bijlmakers MJ. Lck is a key target of imatinib and dasatinib in T-cell activation. Leukemia. 2010;24(4):896–900. Epub 2010/02/12. 10.1038/leu.2010.11 . 20147973

[83] Weichsel R, Dix C, Wooldridge L, Clement M, Fenton-May A, Sewell AK, et al Profound inhibition of antigen-specific T-cell effector functions by dasatinib. Clin Cancer Res. 2008;14(8):2484–91. Epub 2008/04/17. 10.1158/1078-0432.CCR-07-4393 . 18413841

[84] Schade AE, Schieven GL, Townsend R, Jankowska AM, Susulic V, Zhang R, et al Dasatinib, a small-molecule protein tyrosine kinase inhibitor, inhibits T-cell activation and proliferation. Blood. 2008;111(3):1366–77. Epub 2007/10/27. 10.1182/blood-2007-04-084814 17962511 PMC2214733

[85] Shan L, Deng K, Gao H, Xing S, Capoferri AA, Durand CM, et al Transcriptional Reprogramming during Effector-to-Memory Transition Renders CD4(+) T Cells Permissive for Latent HIV-1 Infection. Immunity. 2017;47(4):766–75 e3. Epub 2017/10/19. 10.1016/j.immuni.2017.09.014 29045905 PMC5948104

[86] Pierson T, McArthur J, Siliciano RF. Reservoirs for HIV-1: mechanisms for viral persistence in the presence of antiviral immune responses and antiretroviral therapy. Annu Rev Immunol. 2000;18:665–708. 10.1146/annurev.immunol.18.1.665 10837072

[87] Persaud D, Pierson T, Ruff C, Finzi D, Chadwick KR, Margolick JB, et al A stable latent reservoir for HIV-1 in resting CD4(+) T lymphocytes in infected children. J Clin Invest. 2000;105(7):995–1003. 10.1172/JCI9006 . 10749578 PMC377486

[88] Finzi D, Hermankova M, Pierson T, Carruth LM, Buck C, Chaisson RE, et al Identification of a reservoir for HIV-1 in patients on highly active antiretroviral therapy. Science. 1997;278(5341):1295–300. 10.1126/science.278.5341.1295 9360927

[89] Chun TW, Finzi D, Margolick J, Chadwick K, Schwartz D, Siliciano RF. In vivo fate of HIV-1-infected T cells: quantitative analysis of the transition to stable latency. Nat Med. 1995;1(12):1284–90. 10.1038/nm1295-1284 . 7489410

[90] Kim YK, Bourgeois CF, Pearson R, Tyagi M, West MJ, Wong J, et al Recruitment of TFIIH to the HIV LTR is a rate-limiting step in the emergence of HIV from latency. EMBO J. 2006;25(15):3596–604. Epub 2006/07/29. 10.1038/sj.emboj.7601248 16874302 PMC1538560

[91] Schiralli Lester GM, Henderson AJ. Mechanisms of HIV Transcriptional Regulation and Their Contribution to Latency. Mol Biol Int. 2012;2012:614120 Epub 2012/06/16. 10.1155/2012/614120 22701796 PMC3371693

[92] Kaczmarek Michaels K, Wolschendorf F, Schiralli Lester GM, Natarajan M, Kutsch O, Henderson AJ. RNAP II processivity is a limiting step for HIV-1 transcription independent of orientation to and activity of endogenous neighboring promoters. Virology. 2015;486:7–14. Epub 2015/09/18. 10.1016/j.virol.2015.08.027 26379089 PMC4679410

[93] Nixon CC, Mavigner M, Sampey GC, Brooks AD, Spagnuolo RA, Irlbeck DM, et al Systemic HIV and SIV latency reversal via non-canonical NF-kappaB signalling in vivo. Nature. 2020;578(7793):160–5. Epub 2020/01/24. 10.1038/s41586-020-1951-3 . 31969707 PMC7111210

[94] Hattori SI, Matsuda K, Tsuchiya K, Gatanaga H, Oka S, Yoshimura K, et al Combination of a Latency-Reversing Agent With a Smac Mimetic Minimizes Secondary HIV-1 Infection in vitro. Front Microbiol. 2018;9:2022 Epub 2018/10/05. 10.3389/fmicb.2018.02022 30283406 PMC6156138

[95] Pache L, Dutra MS, Spivak AM, Marlett JM, Murry JP, Hwang Y, et al BIRC2/cIAP1 Is a Negative Regulator of HIV-1 Transcription and Can Be Targeted by Smac Mimetics to Promote Reversal of Viral Latency. Cell Host Microbe. 2015;18(3):345–53. Epub 2015/09/12. 10.1016/j.chom.2015.08.009 26355217 PMC4617541

[96] Jones J, Rodgers J, Heil M, May J, White L, Maddry JA, et al High throughput drug screening for human immunodeficiency virus type 1 reactivating compounds. Assay Drug Dev Technol. 2007;5(2):181–89. Epub 2007/05/05. 10.1089/adt.2006.040 . 17477827

[97] Wang Z, Li J, Xiao W, Long J, Zhang H. The STAT3 inhibitor S3I-201 suppresses fibrogenesis and angiogenesis in liver fibrosis. Lab Invest. 2018;98(12):1600–13. Epub 2018/09/13. 10.1038/s41374-018-0127-3 . 30206312

[98] Siddiquee K, Zhang S, Guida WC, Blaskovich MA, Greedy B, Lawrence HR, et al Selective chemical probe inhibitor of Stat3, identified through structure-based virtual screening, induces antitumor activity. Proc Natl Acad Sci U S A. 2007;104(18):7391–6. Epub 2007/04/28. 10.1073/pnas.0609757104 17463090 PMC1863497

[99] Schindler M, Munch J, Kutsch O, Li H, Santiago ML, Bibollet-Ruche F, et al Nef-mediated suppression of T cell activation was lost in a lentiviral lineage that gave rise to HIV-1. Cell. 2006;125(6):1055–67. Epub 2006/06/17. 10.1016/j.cell.2006.04.033 . 16777597

[100] Tyagi M, Karn J. CBF-1 promotes transcriptional silencing during the establishment of HIV-1 latency. EMBO J. 2007;26(24):4985–95. Epub 2007/11/17. 10.1038/sj.emboj.7601928 18007589 PMC2140115

[101] Marsden MD, Burke BP, Zack JA. HIV latency is influenced by regions of the viral genome outside of the long terminal repeats and regulatory genes. Virology. 2011;417(2):394–9. Epub 2011/07/23. 10.1016/j.virol.2011.06.024 21777932 PMC3163716

[102] Cheadle C, Vawter MP, Freed WJ, Becker KG. Analysis of microarray data using Z score transformation. J Mol Diagn. 2003;5(2):73–81. Epub 2003/04/23. 10.1016/S1525-1578(10)60455-2 12707371 PMC1907322

[103] Dobin A, Davis CA, Schlesinger F, Drenkow J, Zaleski C, Jha S, et al STAR: ultrafast universal RNA-seq aligner. Bioinformatics. 2013;29(1):15–21. Epub 2012/10/30. 10.1093/bioinformatics/bts635 23104886 PMC3530905

[104] Anders S, Pyl PT, Huber W. HTSeq—a Python framework to work with high-throughput sequencing data. Bioinformatics. 2015;31(2):166–9. Epub 2014/09/28. 10.1093/bioinformatics/btu638 25260700 PMC4287950

[105] Love MI, Huber W, Anders S. Moderated estimation of fold change and dispersion for RNA-seq data with DESeq2. Genome Biol. 2014;15(12):550 Epub 2014/12/18. 10.1186/s13059-014-0550-8 25516281 PMC4302049

