# Supplementary figures and images for: Extensive proteomic and transcriptomic changes quench the TCR/CD3 activation signal of latently HIV-1 infected T cells
Source: PLoS Pathog. 2021 Jan 19;17(1):e1008748. doi: 10.1371/journal.ppat.1008748 (PMC7846126; doi:10.1371/journal.ppat.1008748)

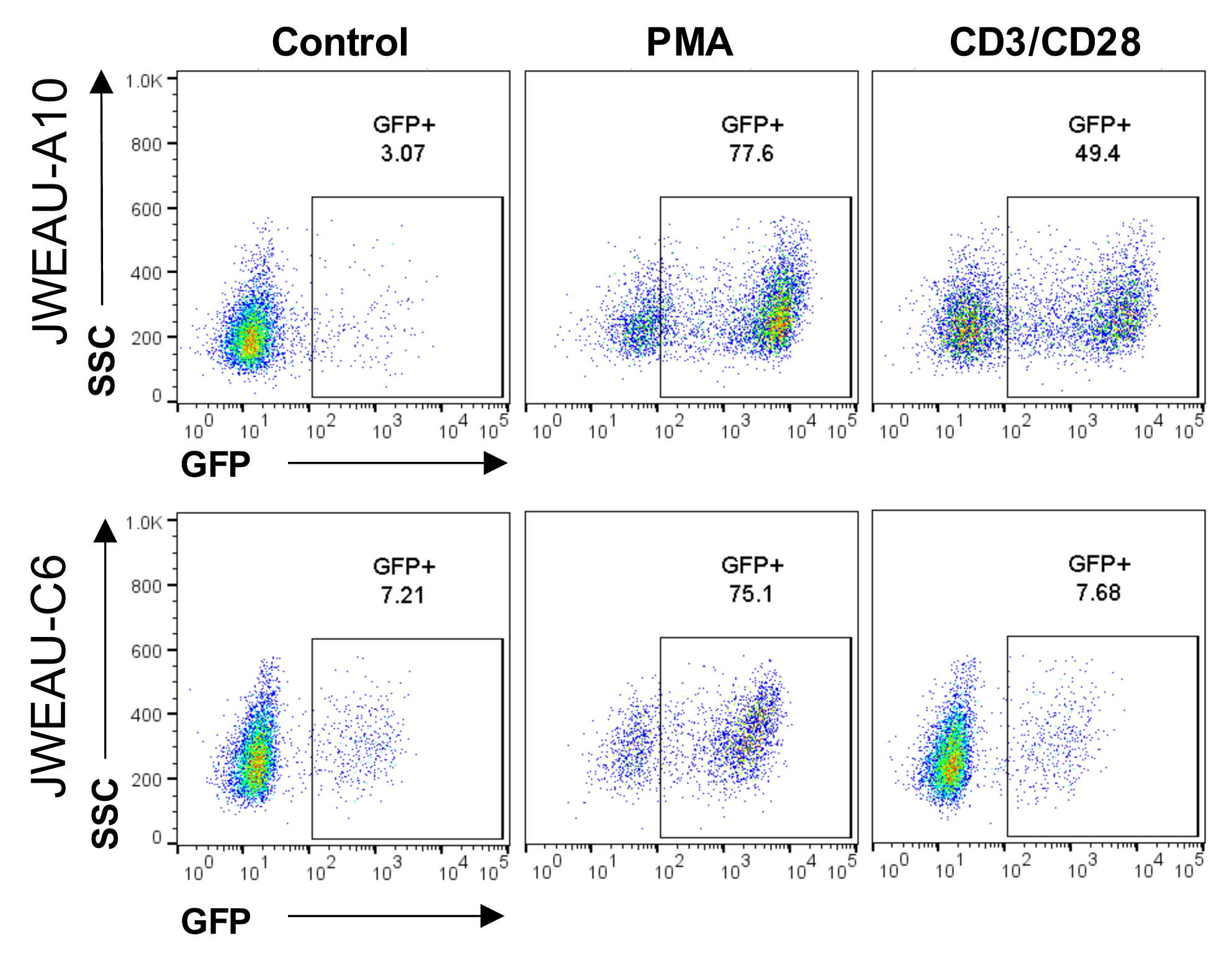

Supplement: S1 Fig — (TIF) [file ppat.1008748.s001.tif]

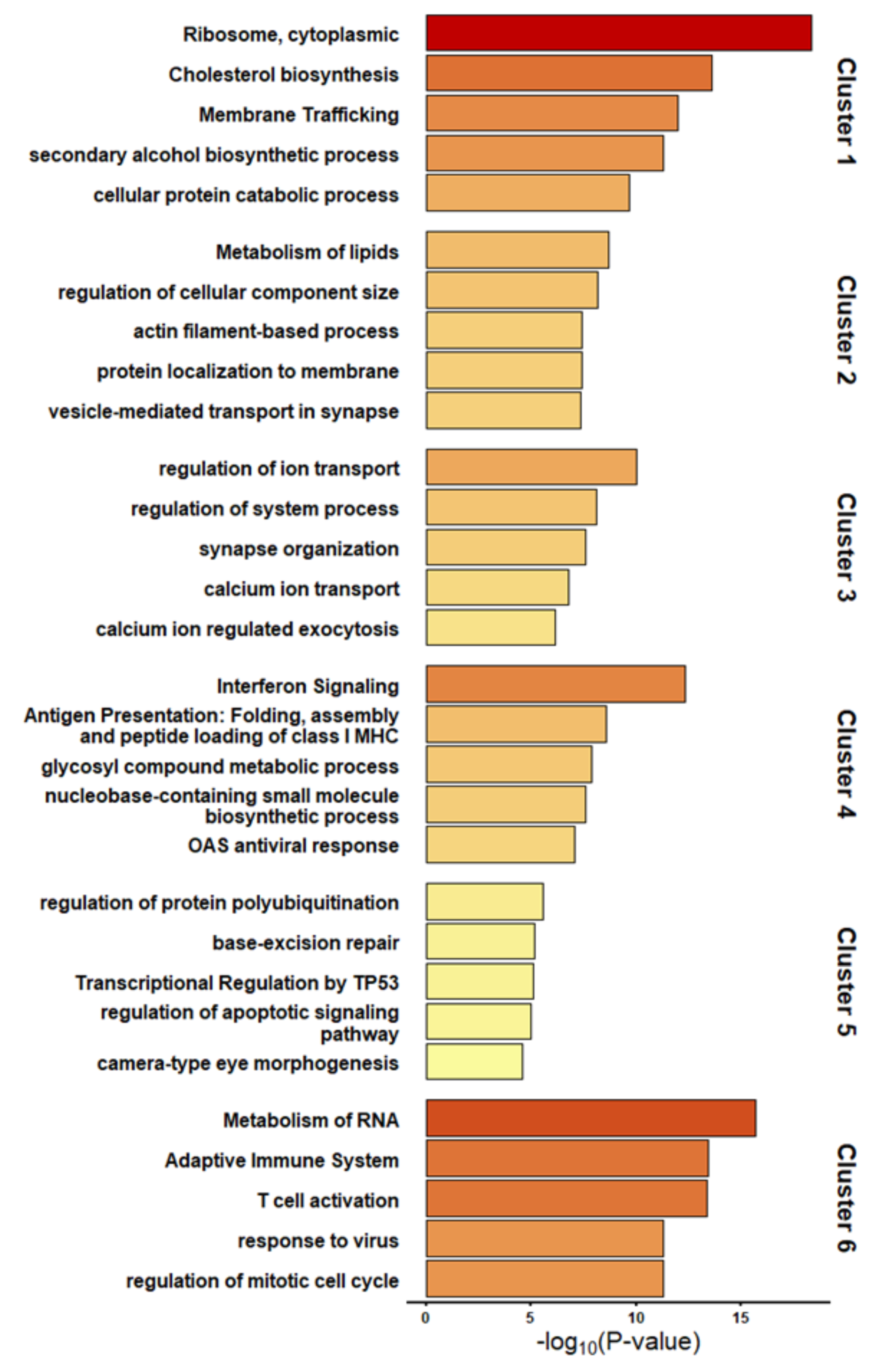

Supplement: S2 Fig — The top 5-ranked motifs for each motif are listed and the p-values for each motif are depicted as histograms. (TIF) [file ppat.1008748.s002.tif]

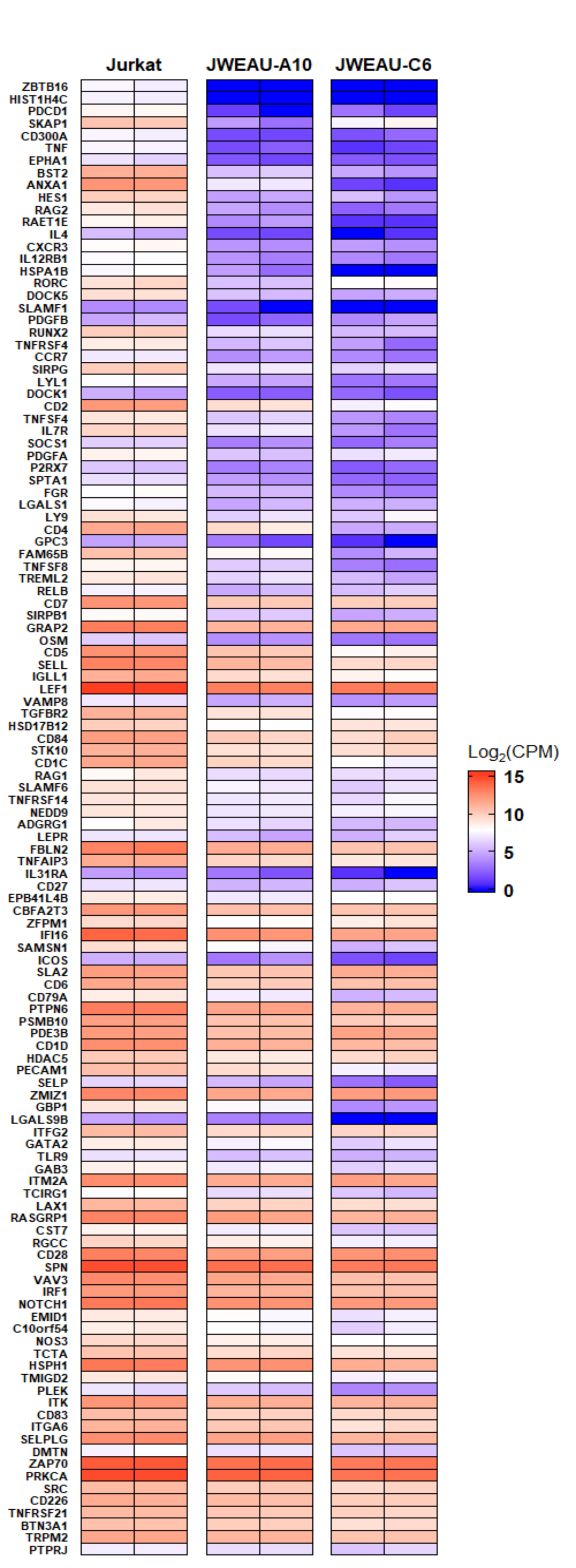

Supplement: S3 Fig — (TIF) [file ppat.1008748.s003.tif]

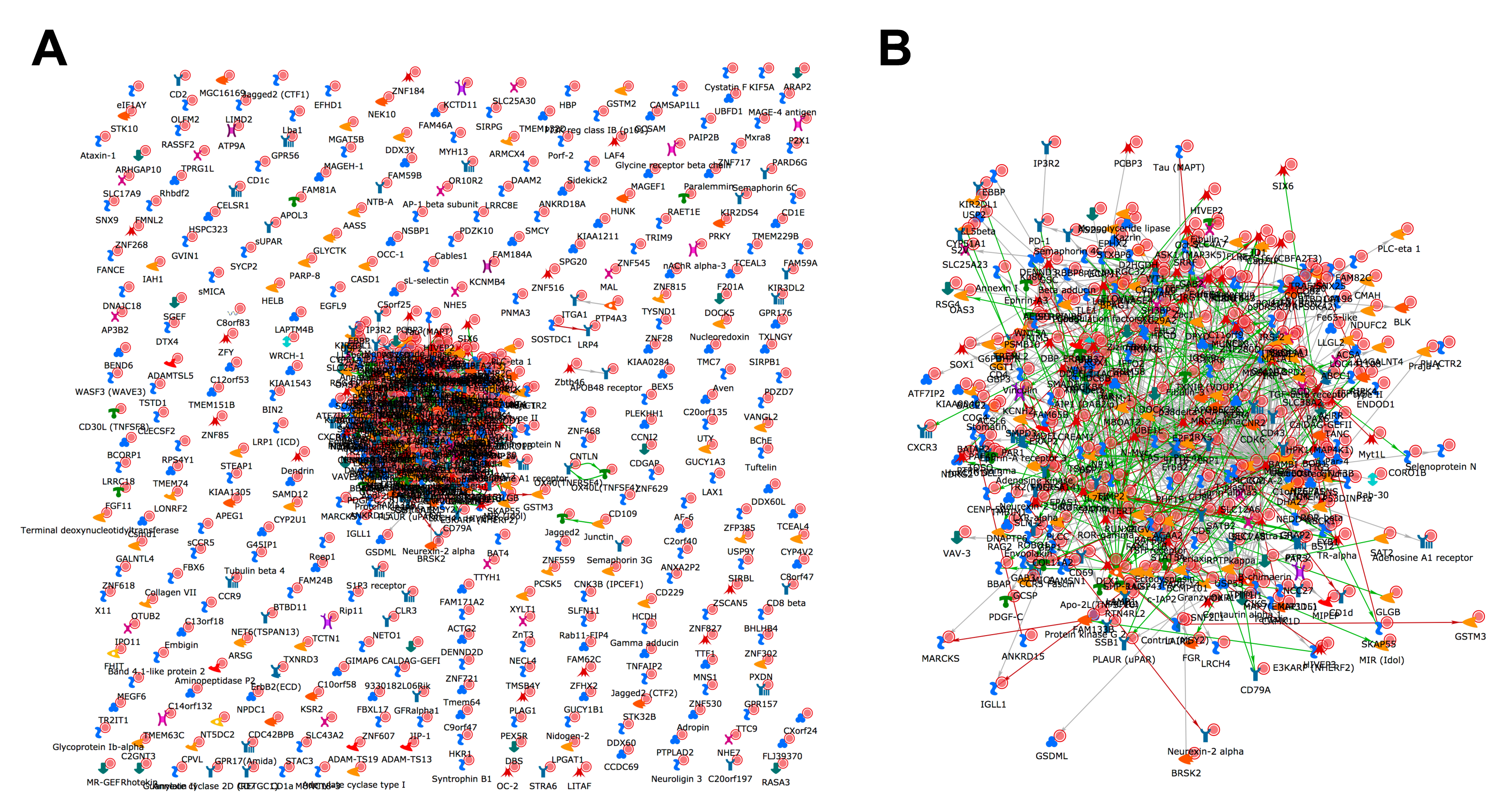

Supplement: S4 Fig — (A) The depiction visualizes the lack of connectivity of a large portion of seed nodes (proteins) around a central interaction network. (B) Visualization of the core network hiding the unconnected genes. (TIF) [file ppat.1008748.s004.tif]

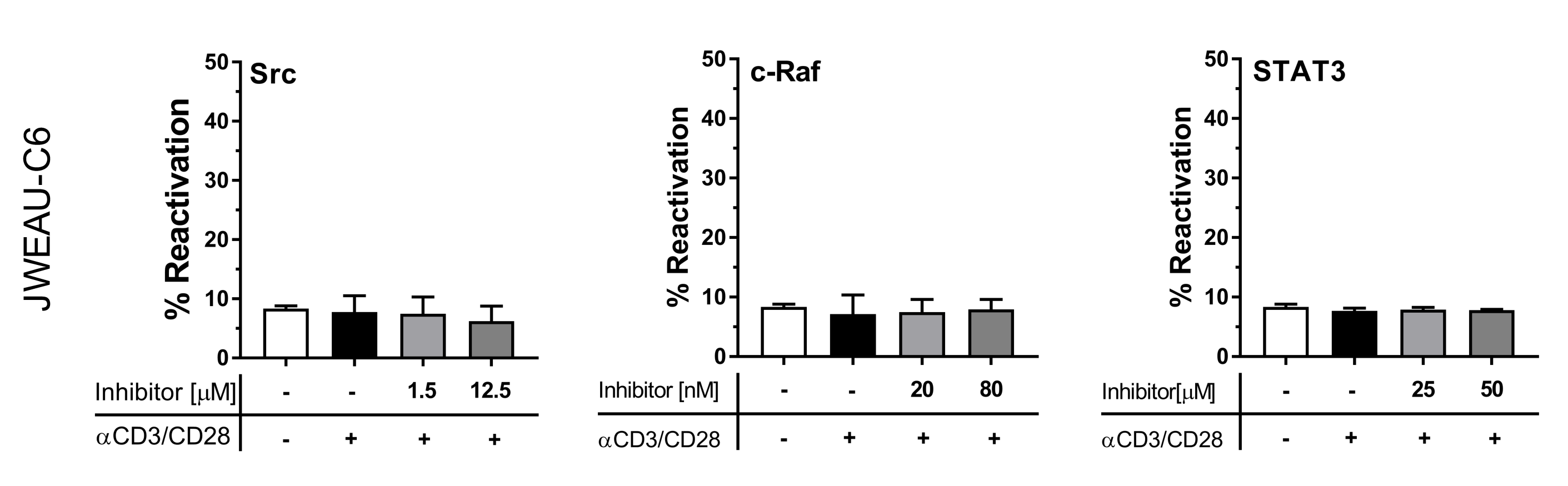

Supplement: S5 Fig — To determine if Src, Raf and STAT3 inhibition would also affect or restore the TCR/CD3 responsiveness of JWEAU-C6 T cells, dasatinib (Src), sorafenib (Raf) or S31-201 (STAT3) were titrated on JWEAU-C6 T cells, which were then stimulated with αCD3/CD28 mAbs. HIV-1 reactivation was determined after 24h by flow cytometric analysis using GFP expression as a surrogate marker of active HIV-1 infection. Data represent the mean ± standard deviation of three independent experiments. (TIF) [file ppat.1008748.s005.tif]

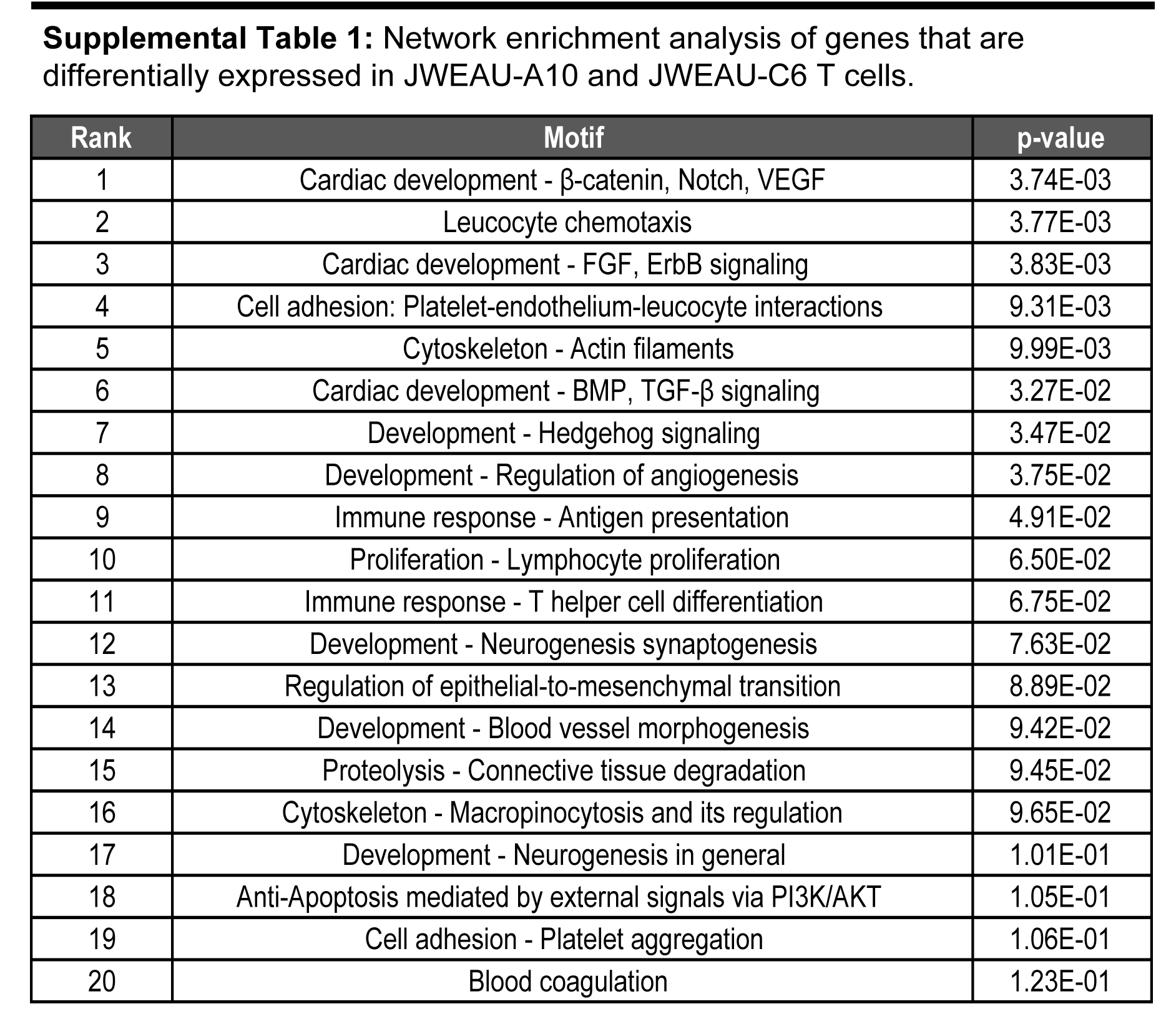

Supplement: S1 Table — (TIF) [file ppat.1008748.s006.tif]
